# Changes in the prevalence of perceived discrimination and associations with probable mental health problems in the UK from 2015 to 2020: A repeated cross-sectional analysis of the UK Household Longitudinal Study

**DOI:** 10.1016/j.ssmph.2024.101667

**Published:** 2024-04-02

**Authors:** Rosanna May Maletta, Michael Daly, Laura Goodwin, Rob Noonan, I Gusti Ngurah Edi Putra, Eric Robinson

**Affiliations:** a Department of Psychology, University of Liverpool, Liverpool, UK; b Department of Psychology, Maynooth University, Co. Kildare, Ireland; c Spectrum Centre for Mental Health Research, Division of Health Research, Lancaster University, Lancaster, UK; d Faculty of Health and Wellbeing, University of Bolton, Bolton, UK

**Keywords:** Discrimination, Mental health, Prevalence trends, Social inequalities, Understanding Society, United Kingdom

## Abstract

**Background:**

Significant social and political changes occurred in the UK between 2015 and 2020. Few studies have examined population level trends in experiencing discrimination and mental health problems during this period.

**Aims:**

To determine prevalence trends in perceived discrimination and probable mental health problems amongst UK adults during 2015–2020.

**Method:**

Repeated cross-sectional data from the UK Household Longitudinal Study was used to estimate nationally representative trends in perceived discrimination and probable mental health problems (GHQ-12; 4+ threshold) among adults between 2015/2016–2019/2020 (25,756 observations). Weighted logistic regression models with post-estimation margins commands determined changes between survey waves controlling for sociodemographic characteristics. Mediation models explored whether changes in perceived discrimination prevalence trends explained trends in probable mental health problems.

**Results:**

From 2015/2016 to 2019/2020 perceived discrimination and probable mental health problems increased significantly by 6·1% (95% CI: 3·4–8·8, *p* <·001) and 4·5% (95% CI: 1·3–7·7, *p* = ·006), respectively. These changes did not tend to reliably differ by sociodemographic grouping. Increased prevalence of probable mental health problems from 2015/2016 to 2019/2020 was partially explained (15·2% of association mediated) by the increase in perceived discrimination observed during the same time period.

**Conclusions:**

Amongst UK adults, the prevalence of perceived discrimination and probable mental health problems increased between 2015/2016 to 2019/2020. Increases in perceived discrimination partially explained increases in probable mental health problems. National measures designed to reduce both discrimination and mental health problems have potential to make substantial improvements to public health and should be prioritised in the UK.

## Highlights

• Prevalence of perceived discrimination increased between 2015 and 2020 in UK adults.
• Prevalence of probable mental health problems also increased during this period.
• Increases in discrimination in part explained increases in mental health problems.
• UK social and political events during this period may have contributed to changes.
• Limited evidence that existing social inequalities significantly reduced over time.

## 1 Introduction

Discrimination is defined as acting less favourably towards an individual due to a personal protected characteristic (e.g. sex, age, race; Equality Act, 2010). Reports of experiencing discrimination (‘perceived discrimination experiences’) are cross-sectionally associated with a range of negative outcomes, including experiencing mental health problems and lower wellbeing (Pascoe & Smart Richman, 2009; Schmitt, Branscombe, Postmes, & Garcia, 2014). Moreover, perceived discrimination experiences are prospectively associated with an increased risk of experiencing psychological distress and the development of depression (Clausen, Rugulies, & Li, 2022; Hackett, Steptoe, & Jackson, 2019; Hackett, Ronaldson, Bhui, Steptoe, & Jackson, 2020; Hackett, Steptoe, Lang, & Jackson, 2020).

In the US, there is evidence that perceived discrimination experiences may have become more common during the last 30 years. For example, a rising trend in several forms of perceived discrimination (e.g. age discrimination) was found between 1995–1996 to 2004–2006 for US adults aged 35–74 (Andreyeva, Puhl, & Brownell, 2008). More recently, between 2004–2005 to 2012–2013 there were significant increases in reported discrimination experiences among adult US Latinos in one study (Cobb et al., 2021).

Since 2015, there have been significant social and political changes in the UK which may have influenced the prevalence of perceived discrimination experiences and in doing so also impacted on population level mental health. This includes changes to welfare legislation (2015–2016; Welfare Reform and Work Act, 2016), the UK's exit from the European Union ‘Brexit’ (2016–2020), and most recently the COVID-19 pandemic (March 2020). Some studies have suggested potential increases in perceived discriminatory experiences as a result of these social and political events. For example, 49% of a sample of young Eastern Europeans reported they had perceived increased racism after the EU referendum (Sime et al., 2017). Moreover, increased racial discrimination experiences after the COVID-19 pandemic began are reported for certain ethnic groups (Ellingworth, Bécares, Šťastná, & Nazroo, 2023; Wong-Padoongpatt, Barrita, & King, 2022). These studies focus on changes in racial discrimination only, and no studies have examined population level UK trends in perceived discrimination experiences more broadly during this period of social and political change. Moreover, a systematic review suggested that UK mental health worsened during the early pandemic (Robinson, Sutin, Daly, & Jones, 2022). Given that perceived discrimination experiences may increase the risk of poorer mental health (e.g. via stress response or health behaviour changes; Pascoe & Smart Richman, 2009), there is a lack of research examining the extent to which changes in population prevalence of perceived discrimination experiences may explain recently observed increases in population level mental health problems among UK adults.

Additionally, it is known that experiences of discrimination and mental health problems vary by sociodemographic grouping (Abrams, Swift, & Houston, 2018; Baker, 2020; Maletta et al., 2023), such as socioeconomic status (SES). As there are inequalities in relation to the likelihood of experiencing each outcome, it is possible that there may also be inequalities within trends (i.e. certain sociodemographic groupings may show a significantly greater increase in perceived discrimination or mental health over time). For example, when assessing changes in racial discrimination during the pandemic in US adults, greater increases are reported for Asian participants, compared to Latinx and White participants (Wong-Padoongpatt et al., 2022). Moreover, in a meta-analysis it is suggested that females showed a greater increase in distress during the pandemic, compared to males (Patel et al., 2022). As there is evidence that changes in the prevalence of perceived discrimination and mental health may vary by sociodemographic grouping, it will also be important to consider sociodemographic differences when exploring population level trends.

To address these questions, we used recent data from the UK Household Longitudinal Study (UKHLS). We focused on 2015–2020, to allow us to assess changes during this period of social and political change in the UK. Moreover, most past research assessing discrimination and mental health explores a specific form of discrimination (e.g. racial discrimination; Hackett, Ronaldson, et al., 2020; Karlsen, Nazroo, McKenzie, Bhui, & Weich, 2005; Wallace, Nazroo, & Bécares, 2016). However, it is well recognised that other forms of discrimination (e.g., gender, age) may contribute to worse mental health (Hackett et al., 2019; Jackson, Hackett, & Steptoe, 2019) and at the population level, overall trends in frequency of all forms of discrimination experiences may therefore be better placed to explain overall trends in population level mental health. Therefore, in the present study we examine population level trends in the prevalence of perceived personal discrimination experiences broadly, and do not restrict trends to a specific type of discrimination experience. We examined nationally representative trends for the prevalence of perceived discrimination and probable mental health problems amongst UK adults between 2015/2016 to 2019/2020. We also tested whether any changes in the prevalence of perceived discrimination in part explained changes in mental health problems during the same period. Additionally, we examined if changes over time for both outcomes differed by sociodemographic characteristics (sex, age, ethnic group, religiousness, health status, income, education, and occupation).

## 2 Method

### 2.1 Sample

The UKHLS (University of Essex, Institute for Social and Economic Research, 2022) is an annual population survey which began in 2009/2010. In the present research we included participants from UKHLS general population comparison and ethnic minority boost samples with available data on a measure of perceived discrimination. Survey response rates, sampling, and COVID-19 survey changes (e.g., the suspension of in person data collection in March 2020) are described in detail elsewhere (Carpenter, 2021; Lynn, 2009; Lynn, Nandi, Parutis, & Platt, 2017; Understanding Society, n.d.). We used a repeated cross-sectional design and focused on waves 7 (2015/2016), 9 (2017/2018), and 11 (2019/2020). We selected these three waves, as the dates are relevant a period of substantial social and political change in the UK and the same sample types are assessed in all three waves (e.g. they include the additional immigrant and ethnic boost sample) which improves consistency of the sample across waves. In addition, this approach enabled us to use the same survey weighting approach across waves to produce nationally representative prevalence estimates of perceived discrimination and probable mental health problems consistently. For full information on survey weighting, see Supplementary A. Data collection for the UKHLS was approved by The University of Essex Ethics Committee, with oral or written consent being taken. As we used secondary anonymised data for analyses, we did not require further ethical approval. The protocol for the study was pre-registered on OSF before statistical analyses were conducted (https://osf.io/mk5nr/).

### 2.2 Measures

#### 2.2.1 Perceived discrimination

Participants reported (yes vs. no) if they felt unsafe, avoided places, were insulted, or attacked during the last 12 months within example locations (e.g., public transport, see Supplementary A for full list), and participants who selected yes were then asked to allocate reason(s) for the experience from: 1) sex, 2) age, 3) ethnicity, 4) sexual orientation, 5) health/disability, 6) nationality, 7) religion, 8) language/accent, 9) dress/appearance. Participants could also select ‘other’ or ‘none of the above’ from this list and as in previous research (Maletta et al., 2023) we did not classify either of these responses as perceived discrimination as the experience was unrelated to a personal, social, or protected characteristic. Participants were classified as having perceived discrimination experiences (binary) if they responded yes to any of the questions described above.

#### 2.2.2 Probable mental health problems

Participants completed the General Health Questionnaire (GHQ)-12. This involved responding to 12 questions (e.g. over the last few weeks, have you recently lost much sleep over worry?). Participants responded on a 4-point scale related to how often the event occurs (e.g. more so than usual). Responses were then recoded into binary variables and summed to create a score of 0–12 for recent mental health (higher scores indicate worse mental health). Scores of 4+ were classed as indicating probable mental health problems, consistent with previous research (Goldberg et al., 1997; Maletta et al., 2023; Morris & Earl, 2017) and studies indicate this threshold in UK samples has high sensitivity and specificity (Goldberg et al., 1997).

#### 2.2.3 Sociodemographic characteristics

Sociodemographic variables included were: sex (male, female), age (16–34, 35–50, 51–64, 65+), ethnic group (White, Mixed, Asian, Black, Other), religiousness (belongs to a religion or not), health (longstanding illness/disability or not), equivalised household income per month (quintiles for each wave, lowest = Category 1 to highest = Category 5), education level (university degree, high school, other/no qualification), and current job (management/professional, intermediate, routine, not in paid employment in the last week).

### 2.3 Statistical analyses

IBM SPSS Statistics v27 was used to pre-process data and analyses were conducted in Stata v17. As missing data was minimal for main variables of interest (see Table S2), complete case analysis was conducted. For further details on missing cases, see Supplementary A. Analyses were weighted to account for survey design (using *svy* commands).

To assess changes in perceived discrimination and probable mental health problems over time, weighted logistic regression models were conducted, with sociodemographic characteristics included as covariates. Post-estimation predicted probabilities (*margins*) were explored, with differences between survey waves being assessed using *lincom.* Alpha was set as <0·05.

Next, to explore if there were sociodemographic characteristic differences in wave-by-wave changes, interaction terms between survey wave and each sociodemographic variable were examined. For descriptive purposes we also report change in outcomes between survey waves by each sociodemographic subgroup. To correct for the large number of sociodemographic comparisons, we adjusted the *p*-values using the Benjamini-Hochberg method (see Supplementary A).

To assess if changes over time were similar for specific types of perceived discrimination, we repeated the above analyses with four common individual types of perceived discrimination (sex, age, ethnicity [including ethnicity, nationality, or language/accent], and health/disability) as separate outcomes in supplementary analyses. Relevant sociodemographic differences in changes were also explored (i.e. sex differences in the change in perceived discrimination due to sex).

Exploratory mediation models were conducted using the *khb* command (Kohler, Karlson, & Holm, 2011) to examine if changes in probable mental health problems between the first and last survey wave were statistically explained (‘mediated’) by changes in perceived discrimination. Mediation models controlled for sociodemographic characteristics as covariates in the main analysis, and were unadjusted in supplementary analyses. See Supplementary A for conditions required for mediation and full information on analytic strategy.

### 2.4 Additional analyses

As Wave 11 (2019/2020) included data collected before and after WHO declared COVID-19 a pandemic, to better understand the potential impact of COVID-19 on estimates we repeated the main analysis adjusting for date of data collection in Wave 11 (i.e. pre vs. post pandemic). As standard survey weights for UKHLS had to be corrected for use across waves examined in the present study (see Supplementary A), we also explored whether results were consistent in the absence of survey weighting.

## 3 Results

### 3.1 Sample characteristics

Overall samples contained 10,264 participants in 2015/2016, 8360 participants in 2017/2018, and 7132 participants in 2019/2020. Main analyses (2015/2016 vs 2019/2020) comprised of participants in these waves (17,396 participants). For sample characteristics in full, see Table S1. For details of missing cases, see Table S2. For weighted prevalence of perceived discrimination and probable mental health problems by sociodemographic subgroup and survey wave, see Table 1.

**Table 1:** Prevalence of perceived discrimination and probable mental health problems by sociodemographic subgroup and survey wave.

**Population Subgroup**	**Perceived Discrimination**^a^			**Probable Mental Health Problems**^b^		
	Wave 7 (2015/2016)	Wave 9 (2017/2018)	Wave 11 (2019/2020)	Wave 7 (2015/2016)	Wave 9 (2017/2018)	Wave 11 (2019/2020)
	**Weighted *n***& %	**Weighted*****n*** & %	**Weighted *n*** & %	**Weighted *n*** & %	**Weighted *n*** & %	**Weighted*****n*** & %
**Overall**	**9371**	**7829**	**6757**	**9196**	**7206**	**6377**
	14·6%	18·2%	20·1%	17·9%	17·7%	22·1%
**Sex**	**9370**	**7828**	**6755**	**9195**	**7205**	**6375**
Male	8·9%	12·1%	13·4%	14·7%	13·4%	17·3%
Female	21·1%	24·9%	27·5%	21·5%	22·3%	27·5%
**Age**	**9370**	**7829**	**6757**	**9195**	**7206**	**6377**
16-34	20·3%	29·2%	25·6%	23·5%	21·0%	26·0%
35-50	13·2%	13·8%	15·6%	17·7%	20·7%	25·7%
51-64	9·2%	11·9%	20·3%	14·3%	15·5%	19·4%
65+	11·9%	13·1%	17·0%	11·8%	10·5%	16·3%
**Ethnic Group**	**9312**	**7781**	**6718**	**9139**	**7164**	**6342**
White	12·3%	16·7%	18·5%	16·8%	16·6%	21·9%
Mixed	34·2%	22·3%	38·8%	26·9%	30·9%	34·9%
Asian	23·4%	24·2%	32·1%	21·3%	19·1%	21·4%
Black	24·2%	24·9%	21·5%	22·3%	16·6%	19·8%
Other	24·0%	43·8%	23·7%	20·2%	37·8%	16·5%
**Religious (Identifies as belonging to a religion)**	**9080**	**7752**	**6633**	**8883**	**7103**	**6262**
Yes	15·1%	17·0%	19·5%	17·1%	15·6%	19·0%
No	13·5%	19·3%	20·4%	18·8%	19·1%	25·2%
**Health (Has longstanding illness/disability)**	**9355**	**7818**	**6724**	**9184**	**7199**	**6352**
Yes	18·6%	22·0%	27·6%	24·1%	28·8%	29·5%
No	12·5%	16·2%	15·9%	14·8%	11·9%	18·2%
**Equivalised Household Income per Month**	**9270**	**7589**	**6501**	**9098**	**6985**	**6143**
Category 1 *(lowest)*	13·8%	17·6%	23·0%	21·2%	27·6%	26·9%
Category 2	17·4%	19·1%	14·5%	21·8%	21·5%	24·2%
Category 3	20·2%	22·8%	25·1%	22·9%	21·3%	23·3%
Category 4	12·3%	18·0%	21·1%	13·5%	15·4%	21·5%
Category 5 (*highest)*	11·6%	14·4%	17·8%	15·9%	11·3%	19·1%
**Education Level**	**8662**	**7276**	**6410**	**8420**	**6769**	**6048**
Other/No Qualification	15·4%	19·6%	15·0%	17·9%	22·1%	17·1%
High School Qualification	17·5%	20·2%	23·6%	20·3%	18·2%	23·1%
University Degree	11·8%	16·0%	18·3%	16·0%	15·9%	22·6%
**Current Job**	**9275**	**7606**	**6482**	**9107**	**6995**	**6112**
Not in paid employment in the last week	16·8%	19·7%	21·3%	21·9%	19·9%	23·4%
Routine	17·2%	20·5%	21·1%	17·5%	18·1%	19·2%
Intermediate	13·9%	23·2%	20·0%	14·3%	15·8%	16·6%
Management & Professional	10·4%	11·3%	18·5%	14·7%	14·8%	25·6%

Notes.

a Perceived discrimination = self-reported feeling unsafe, avoiding places, being insulted, or being attacked due to a personal characteristic.

b Probable mental health problems = GHQ-12 score of 4+.

### 3.2 Changes in perceived discrimination

**Fig. 1:**
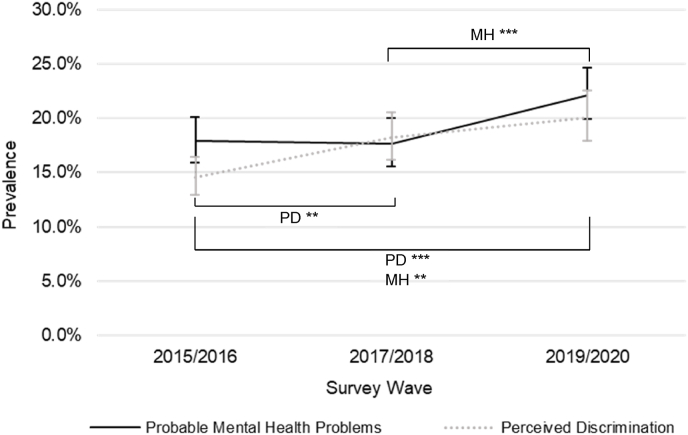
Weighted Prevalence of Perceived Discrimination and Probable Mental Health Problems by Survey Wave. PD = significant change in perceived discrimination in models which controlled for all sociodemographic characteristics. MH = significant change in probable mental health problems in models which controlled for all sociodemographic characteristics. *p <·05; **p <·01; ***p <·001.

Prevalence of perceived discrimination increased from 14·6% in 2015/2016 to 20·1% in 2019/2020 (see Fig. 1). Between 2015/2016 to 2019/2020 perceived discrimination increased significantly by 6·1% (95% CI: 3·4–8·8, *p* <·001). Between these waves, from 2015/2016 to 2017/2018 there was a 4·0% significant increase (95% CI: 1·3–6·6, *p* = ·003) and the 2·3% increase in perceived discrimination between 2017/2018 to 2019/2020 was non-significant (95% CI: −0·6 to 5·2, *p* = ·119).

### 3.3 Changes in probable mental health problems

Prevalence of probable mental health problems was similar in 2015/2016 (17·9%) and 2017/2018 (17·7%), but increased to 22·1% in 2019/2020 (see Fig. 1). Between 2015/2016 to 2019/2020 probable mental health problems increased significantly by 4·5% (95% CI: 1·3–7·7, *p* = ·006). Between these waves, from 2015/2016 to 2017/2018 there was a non-significant 0·9% decrease in probable mental health problems (95% CI: −3·8 to 2·0, *p* = ·548) and between 2017/2018 to 2019/2020 there was a 5·3% significant increase (95% CI: 2·4–8·2, *p* <·001).

The overall sample changes in perceived discrimination and probable mental health problems from 2015/2016 to 2019/2020 analysis were the same in unweighted analyses (see Supplementary C).

### 3.4 Differences between sociodemographic groups in the changes in perceived discrimination and probable mental health problems

There was no statistical evidence that survey wave interacted with any of the sociodemographic characteristics for perceived discrimination or probable mental health problems (see Table 2), indicating that size of weighted changes from 2015/2016 to 2019/2020 were not meaningfully different across sociodemographic groups. We did not find any evidence that weighted changes from 2015/2016 to 2017/2018 and weighted changes from 2017/2018 to 2019/2020 differed by sociodemographic group (see Table S6). See Table S7 for changes by each sociodemographic subgroup. However, when these models were repeated unweighted, some evidence of significant interactions by sociodemographic groupings were found that were consistent with the non-significant trends in weighted analyses (e.g. those with a longstanding disability exhibited a greater increase in perceived discrimination from 2015/2016 to 2019/2020; see Table S3 for full details).

**Table 2:** Differences between sociodemographic groups in the changes in perceived discrimination and probable mental health problems from 2015/2016 to 2019/2020.

**Population Subgroup**	**Difference between Subgroups in the Changes in Perceived Discrimination from 2015/2016 to 2019/2020**^a^		**Difference between Subgroups in the Changes in Probable Mental Health Problems from 2015/2016 to 2019/2020**^b^	
	%	(95% CI)	%	(95% CI)
**Sex (Comparison: Males)**				
Females	+1·7	(−4·0 to 7·4)	+3·9	(−2·5 to 10·4)
**Age Group (Comparison: 16–34 Years)**				
35–50 Years	−4·1	(−11·0 to 2·7)	+5·8	(−5·3 to 16·8)
51–64 Years	+4·9	(−2·9 to 12·7)	+3·2	(−6·1 to 12·5)
65+ Years	−1·4	(−9·2 to 6·5)	+1·2	(−7·3 to 9·6)
**Ethnic Group (Comparison: White Respondents)**				
Mixed	−2·0	(−19·0 to 14·9)	+0·8	(−13·1 to 14·7)
Asian	+1·5	(−6·5 to 9·5)	−5·5	(−13·3 to 2·4)
Black	−10·4	(−22·7 to 2·0)	−9·1	(−18·9 to 0·6)
Other	−11·0	(−26·6 to 4·6)	−9·5	(−36·8 to 17·7)
**Religious (Comparison: Does not identify as belonging to a religion)**				
Identifies as belonging to a religion	−3·4	(−9·5 to 2·6)	−3·3	(−10·4 to 3·7)
**Health (Comparison: Does not have a longstanding illness/disability)**				
Has a longstanding illness/disability	+5·0	(−2·6 to 12·5)	+0·4	(−6·8 to 7·7)
**Equivalised Household Income Per Month (Comparison: Category 1)**				
Category 2	−11·6	(−21·8 to −1·4)	−1·6	(−13·5 to 10·3)
Category 3	−5·1	(−16·6 to 6·3)	−3·0	(−14·4 to 8·5)
Category 4	+2·2	(−7·8 to 12·2)	+4·5	(−6·3 to 15·3)
Category 5	+0·3	(−9·8 to 10·4)	+0·5	(−9·2 to 10·2)
**Education Level (Comparison: Other/No Qualification)**				
High School Qualification	+6·7	(−2·3 to 15·7)	+1·8	(−7·0 to 10·6)
University Degree	+7·0	(−1·8 to 15·7)	+5·9	(−2·8 to 14·6)
**Current Job (Comparison: Not in Paid Employment in the Last Week)**				
Routine	+0·4	(−8·3 to 9·1)	+0·2	(−10·0 to 10·3)
Intermediate	−2·1	(−10·0 to 5·8)	+0·4	(−8·1 to 8·9)
Management & Professional	+4·7	(−2·3 to 11·8)	+8·1	(0·5 to 15·7)

Note: Interaction terms were added to multivariable models containing all sociodemographic characteristics as covariates. *P*-values are adjusted using the Benjamini-Hochberg method. Differences displayed are lincom differences between the changes in predicted probabilities between waves (marginal effects), multiplied by 100 to get percentage point differences. The difference in the changes are between the displayed comparison group and non-comparison group (e.g. males vs. females). Negative values indicate that the change for the comparison group was greater than the change for the non-comparison group. For details of the changes in the outcomes between waves by subgroup, see Table S7.

a Perceived discrimination weighted *n* = 14,172. Unweighted *n* = 14,281.

b Probable mental health problems weighted *n* = 13,713. Unweighted *n* = 13,731.

### 3.5 Analyses examining the relationship between increases in perceived discrimination and probable mental health problems

Among participants from the 2015/2016 and 2019/2020 waves, the prevalence of probable mental health problems was 11·5% (95% CI: 7·0–16·0, p <·001) higher among participants that reported perceived discrimination (29·1%), compared to participants that did not (17·6%). The association between perceived discrimination experiences and having probable mental health problems was similar in 2015/2016 and 2019/2020 (see Supplementary E), suggesting that any direct association between experiencing discrimination and mental health problems had not changed in size over time. Next, in formal mediation analyses we found that the change in prevalence of probable mental health problems observed between 2015/2016 to 2019/2020 was partially mediated (15·2% of association) by the observed increase in prevalence of perceived discrimination during the same time period (see Table 3). Similar results were found when models were repeated unadjusted for sociodemographic characteristics (see Table S8).

**Table 3:** Mediation model exploring perceived discrimination experiences as a mediator of the association between survey wave and probable mental health problems.

**IV**	**Mental Health**		**Effect Ratio**
	B	95% CIs	
**Survey Wave (Reference: Wave 7 [2015/2016])**			
Total effect of Wave (Wave 11 [2019/2020])	0·29*	0·07 to 0·51	
Direct effect of Wave (Wave 11 [2019/2020])	0·24*	0·03 to 0·46	
*Indirect effect* via *perceived discrimination*	0·04**	*0·02 to 0·07*	*15·2%*

Note: *n* = 10,949 (unweighted *n* = 13,229). Model controls for all sociodemographic characteristics (adjusted model).

*p <·05; **p <·01; ***p <·001.

### 3.6 Further analysis

#### 3.6.1 Discrimination type

We repeated primary perceived discrimination analyses on individual reasons for discrimination (i.e., due to sex, due to age, due to any ethnicity reasons, due to health/disability). Probability of perceiving personal discrimination due to health/disability significantly increased between 2015/2016 and 2019/2020 by 9·8% (95% CI: 3·4–16·3, *p* = ·003), whereas perceived discrimination based on sex (+2·3%, 95% CI: −3·4 to 7·9, *p* = ·430), age (+4·7%, 95% CI: −3·3 to 12·7, *p* = ·252) and ethnicity (−0·4%, 95% CI: −7·0 to 6·2, *p* = ·911) did not statistically significantly change. We then assessed if these changes differed by relevant sociodemographic groups. For example, we assessed whether the change in perceived discrimination due to sex differed by sex, perceived discrimination due to age differed by age group, perceived discrimination due to ethnicity differed by ethnic group, and perceived discrimination due to health/disability differed by health status. However, we did not find any evidence that these changes differed by the sociodemographic groups tested (see Supplementary G).

#### 3.6.2 COVID-19 adjustment

For full COVID-19 adjusted results, see Supplementary H. Assessing interview dates revealed that 53·0% of the weighted sample in 2019/2020 was collected pre-COVID and 47·0% post. Post COVID-19 participants in 2019/2020 had higher prevalence of probable mental health problems by 5·5%, relative to pre-COVID-19 2019/2020.

We controlled for COVID-19 effects in primary analyses by repeating analyses while including interview start date (before or after COVID-19) as a covariate. Controlling for this variable, resulted in the 2015/2016 to 2019/2020 change in probable mental health problems becoming non-significant (+2·1%, 95% CI: −1·7 to 5·9, *p* = ·282), suggesting that observed increases in mental health observed over the study period primarily occurred after the outbreak of COVID-19. Perceived discrimination did not differ according to when in 2019/2020 data was collected, and the change between 2015/2016 to 2019/2020 in perceived discrimination remained significant (+6·9%, 95% CI: 3·5–10·4, *p* <·001) after adjustment for interview date.

Consistent with the main analysis there was no statistical evidence that survey wave interacted with any of the sociodemographic variables for perceived discrimination or probable mental health problems after controlling for COVID-19 (see Table S12).

Controlling for COVID-19 revealed further consistent results. For example, probable mental health problems were significantly greater by 11·6% (95% CI: 7·1–16·2, *p* <·001) for those who had perceived discrimination, compared to those who had not. Moreover, the size of the change in probable mental health problems between 2015/2016 and 2019/2020 was not significantly different for those who did and did not perceive discrimination (see Supplementary H for full details).

## 4 Discussion

In this repeated cross-sectional analysis study of UK adults, we found that the prevalence of perceived discrimination and probable mental health problems increased significantly between 2015/2016 to 2019/2020. The increase in probable mental health problems became non-significant after controlling for COVID-19, suggesting observed increases to probable mental health problems primarily occurred after the COVID-19 outbreak. There was no consistent evidence that the observed increases greatly differed by sociodemographic groupings, relating to sex, age, ethnic group, religiousness, health status, income, education, or occupation. In mediation analyses, the increased prevalence of perceived discrimination during this time period statistically accounted for 15% of the observed increase in probable mental health problems from 2015/2016 to 2019/2020. Our study is the first to evidence population level changes in the prevalence of perceived discrimination amongst UK adults during a period of significant social and political change (2015–2020).

The finding that the prevalence of probable mental health problems increased from 2015/2016 to 2019/2020 is consistent with other reports of an increase in mental health problems among UK adults in recent years after the outbreak of COVID-19 (e.g. Robinson et al., 2022). Our results indicated that these increases did not greatly differ between sociodemographic groups, suggesting that there was no evidence that existing baseline social inequalities significantly reduced over time. This is consistent with past research demonstrating that gender inequalities in common mental health disorders were present over time in England (1993–2014; Baker, 2020), and recent estimates demonstrating social inequalities in probable mental health problems among UK adults (Maletta et al., 2023). In line with this, in the present study we found that the prevalence of both perceived discrimination and probable mental health problems were consistently higher among women and younger adults, whilst reporting of discrimination experiences remained persistently higher in non-white ethnic groups (Abrams et al., 2018; Maletta et al., 2023).

The association between perceived discrimination experiences and poorer mental health is well-evidenced, both cross-sectionally (Pascoe & Smart Richman, 2009; Schmitt et al., 2014) and prospectively in analyses centred on individual level change (Clausen et al., 2022; Hackett et al., 2019; Hackett, Ronaldson, et al., 2020; Hackett, Steptoe, et al., 2020). In the present research we replicated the previously observed cross-sectional association between perceived discrimination and mental health problems, but did not find statistical evidence that the size of this association differed significantly between 2015/2016 and 2019/2020. However, we conducted a mediation analysis to examine if the change in prevalence of perceived discrimination experiences was associated with the observed increases in prevalence of probable mental health problems. This analysis revealed that perceived discrimination prevalence increases explained approximately 15% of the increase in prevalence of probable mental health problems amongst UK adults from 2015/2016 to 2019/2020.

The time period studied (2015–2020) coincided with a range of social and political events within the UK, including the UK's exit from the European Union or the ‘Brexit’ timeline; the Welfare Reform and Work Act (2016) which resulted in significant changes to social welfare benefits available for households; and the final survey wave coincided with the beginning of the COVID-19 pandemic.

It is plausible that one or more of these events may have contributed to the observed changes in perceived discrimination and probable mental health problems in the UK. Significant increases in perceived discrimination were observed wave-on-wave from 2015/2016 to 2017/2018 and from 2015/2016 to 2019/2020, periods which include the national referendum on the European Union and the subsequent exit process. This event has previously been associated with increases in negative outcomes, such as hate crimes (Carr, Clifton-Sprigg, James, & Vujic, 2020), fear of ethnic and racial harassment (Nandi & Luthra, 2021), and mental distress (Hervy, Cavalli, Madia, & Nicodemo, 2022). Increases in reports of perceived discrimination relating to disability were particularly pronounced in the present study and this may be associated with the 2016 UK welfare reform (Welfare Reform and Work Act, 2016). People with disabilities may have experienced greater negative impacts, as this legislation required many people unable to work due to disability to be reassessed for welfare eligibility (including changes in the eligibility for those living with psychological distress) and subsequent reductions in the welfare support they received (Welsh Government, 2019). Qualitative work has captured the experiences of those with physical disabilities who have experienced benefits changes. These include negative experiences such as facing societal stigma due to claiming benefits, and the harmful impact that both this judgement and other factors (e.g. fear or experiences of financial difficulties due to benefit cuts) has on mental health (Saffer, Nolte, & Duffy, 2018).

Moreover, after controlling for the outbreak of COVID-19 in analyses, the observed increase in perceived discrimination remained significant. This suggests that the increase in perceived discrimination observed from 2015/2016 to 2019/2020 was not solely due to the outbreak of COVID-19. However, some research has found evidence of increases in perceived discrimination experiences during the pandemic (Ellingworth et al., 2023; Wong-Padoongpatt et al., 2022), in addition to specific discrimination experiences attributable to the pandemic (e.g. being threatened/harassed because people think you may have COVID-19; Strassle et al., 2022). Therefore, the COVID-19 pandemic may have in part contributed to increases in discrimination observed in later waves in the present study.

Increases in probable mental health problems were attenuated to non-significance in analyses that controlled for the outbreak of COVID-19, which suggests that the higher prevalence of probable mental health problems observed in the 2019/2020 wave may have been primarily driven by the COVID-19 pandemic. This finding is consistent with a number of studies suggesting that during the early stages of the pandemic mental health symptoms increased (Robinson et al., 2022). Further research examining if the observed upward trends in perceived discrimination and probable mental health problems and the associations between discrimination and mental health (Lee et al., 2022) have persisted beyond 2019/2020 would now be informative.

Moreover, our main analyses explored trends in perceived discrimination broadly, in that domains (e.g. physical assault) or locations (e.g. public places) were not the focus. Much of the previous literature has selected to focus on only one type of discrimination (e.g. the impact of racial discrimination on mental health; Hackett, Ronaldson, et al., 2020; Wallace et al., 2016). We did explore trends in discrimination by type (e.g., sex, age) and analysis revealed significant increases in reports of perceived discrimination due to health/disability, but no significant increases in reports of sex, age, or ethnicity-based discrimination during the same time period. Therefore, future research may also benefit from examining whether specific domains or locations of discrimination experiences have changed or remained consistent over time.

Strengths of the present research include the use of a large longitudinal dataset, which allowed us to estimate representative prevalence of perceived discrimination and probable mental health problems trends for UK adults. Limitations include that the self-report measure of perceived discrimination is reliant on recalling past experiences, which will be prone to recall and interpretation bias. Moreover, although the self-report GHQ-12 is widely used to examine probable mental health problems, it does not provide a clinical diagnosis. The UKHLS oversamples ethnic minority groups, however survey weights were applied to produce nationally representative estimates and ethnicity-based differences in outcomes were accounted for. We used complete cases analyses in the present study, as missing data in the main variables of interest (e.g., perceived discrimination, probable mental health problems) was low. However, younger age groups, some non-White ethnic groups, religious respondents, and lower SES groups had slightly higher percentages of missingness. We also found that a slightly higher percentage (2·7%) of those who did not perceive discrimination had incomplete cases compared to those who did perceive discrimination (see Supplementary A). Therefore, our analyses are more likely to slightly overestimate than underestimate the prevalence of perceived discrimination.

Furthermore, over time survey interview modes have changed, in that a higher proportion of interviews are being completed via the web (instead of via face-to-face interviews) in waves 9 and 11, compared to wave 7. As such it is possible that this change in interview survey mode may have influenced estimates (i.e. perceived discrimination) in later waves. We were unable to conduct weighted analyses on earlier UKHLS waves prior to the main study period and this would have increased confidence in the present study periods findings in relation to earlier historic trends in outcomes. Because we were required to make a correction to standard survey weights for UKHLS we examined if results were consistent in the absence of weights. In the main study period (2015/2016 to 2019/2020) overall trend results were identical in unweighted and weighted analyses. Finally, our analyses are unable to identify the likely causes of changes in prevalence of perceived discrimination and mental health problems. Further research is now warranted to better understand the role that recent social and political events may have had on recent increases in perceived discrimination and mental health problems among the UK adult population.

## 5 Conclusions

Amongst UK adults the prevalence of perceived discrimination and probable mental health problems increased between 2015/2016 to 2019/2020. The increase in perceived discrimination partially explained the increase in probable mental health problems. These findings highlight the importance of considering the role of discrimination when designing policy to reduce population level mental health. National measures to reduce both discrimination and mental health problems would have potential to make substantial improvements to public health and should be prioritised in the UK.

## Data sharing

Data are openly available via the UK Data Service: https://doi.org/10.5255/UKDA-SN-6614-16.

## Funding source

The first author completed this work as part of their PhD, which is supported by an 10.13039/501100000269Economic and Social Research Council NWSSDTP case studentship [grant number: 10.13039/501100018726ES/P000665/1]. The funding source had no involvement in study design, analysis and interpretation of data, writing of the report, and in the decision to submit the article for publication.

## Ethical statement

The UKHLS complies with the ethical standards of the relevant national and institutional committees on human experimentation and with the Helsinki Declaration of 1975, as revised in 2008. All procedures involving human subjects/patients were approved by The University of Essex Ethics Committee, with oral or written consent being taken from participants. As we used secondary anonymised data for analyses, we did not require further approvals.

## CRediT authorship contribution statement

**Rosanna May Maletta:** Conceptualization, Methodology, Formal Analysis, Data Curation, Writing – original draft, Writing – review & editing, Visualization. **Michael Daly:** Conceptualization, Methodology, Writing – review & editing. **Laura Goodwin:** Conceptualization, Writing – review & editing. **Rob Noonan:** Conceptualization, Writing – review & editing. **I Gusti Ngurah Edi Putra:** Conceptualization, Formal Analysis, Validation, Writing – review & editing. **Eric Robinson:** Conceptualization, Methodology, Writing – review & editing, Supervision, Funding acquisition.

## Declaration of competing interest

None.
