# Supplementary material for: Changes in the prevalence of perceived discrimination and associations with probable mental health problems in the UK from 2015 to 2020: A repeated cross-sectional analysis of the UK Household Longitudinal Study
Source: SSM Popul Health. 2024 Apr 2;26:101667. doi: 10.1016/j.ssmph.2024.101667 (PMC11081791; doi:10.1016/j.ssmph.2024.101667)
Supplement: Multimedia component 1 [file mmc1.docx]

# Supplementary Materials

## Contents

A. Additional Method Information (pages 1-3)

B. Sample Characteristics and Missing Cases (pages 4-5)

C. Results of Primary Analyses Using Unweighted Analysis (pages 6-8)

D. Repeated Analyses with Other Wave Comparisons (pages 9-10)

E. Population Subgroup Changes Between Waves (pages 11-12)

F. Unadjusted Mediation Models (page 13)

G. Individual Reasons for Discrimination (pages 14-16)

H. Impact of COVID-19 (pages 17-19)

I. Exploratory Analysis: Exploration of Earlier Pre-Study Waves of UKHLS (pages 20-21)

## A. Additional Method Information

### Additional Sample Information

**Survey Wave Dates**

In each 2-year survey wave of UKHLS a small amount of data is collected outside of the main wave time period. For example, in wave 9 (2017/2018) 9·2% of data was collected from January 2019 to May 2019. To be consistent with previous UKHLS research using the perceived discrimination measure^1–4^ we referred to these waves within the article using the main two years of data collection, e.g. wave 9 (2017/2018).

**Samples**

Ethnic minority boost participants were sampled from households in high-concentration ethnic minority areas. These participants belonged to or had parents/grandparents that belonged to ethnic minority groups. A smaller sample of those in low-density ethnic minority areas was also included. Later, those born outside of the UK were also incorporated as a boost sample. The general population comparison sample is a random sample of 500 households taken from the main sample of UK households. Full sample details are reported elsewhere.^5^

**Survey Weight Correction**

We applied an existing cross-sectional weight, designed for the extra five minutes group of UKHLS to ensure non-biased standard errors and that results were nationally representative. However, applying this weight excluded several participants with perceived discrimination responses (e.g. the immigrant boost sample, some non-ethnic minority respondents), meaning the analysis was attempting to produce nationally representative estimates from a small number of participants. Small samples may be inappropriate to confidently produce nationally representative prevalence estimates. As such we corrected the weight to include additional participants. The existing survey weights^6^ were corrected using the following method, which we discussed and agreed upon with the UKHLS Understanding Society sampling team:

1. Weighted the perceived discrimination question sample or ‘extra five minutes’ sample (e.g. for 2015/2016, *n* = 10,264) using x_indinui_xw.
2. With this weight applied we ran descriptive statistics on a created boosted race variable to get proportions in each of the 8 groups for the ‘extra five minutes’ sample for that wave (sample proportions).

*Boosted race variable categories: 1) Immigrant between 2009-2015 (wave 1-5); 2) Anyone else born outside of the UK; 3) Indian; 4) Caribbean; 5) African; 6) Bangladeshi; 7) Pakistani; 8) Other (includes White British and Other ethnic groups). If they had missing or wild data for one level, they were coded at a lower level e.g. if they had missing data on whether they were born outside the UK, but had data for their ethnic group, they were coded as their ethnic group.*

1. Applied the x_psnenui_xw weight to the full sample (indall file) and obtained the proportions in each of the 8 groups for that wave (population proportions).
2. Divided the produced population proportion by the sample proportion for each of the 8 groups.
3. Produced a weight variable where each of the 8 groups was assigned the number from step 4 (e.g. if the answer from step 4 was 0·1359 for the immigrant between wave 1-6 group then each participant in this group was assigned a weight value of 0·1359). Labelled this variable as: weightnew.
4. Multiplied the standard weight and this new weight variable (x_indinui_xw * weightnew) to produce weightnew2.
5. Compared proportions of the created boosted race variable for the ‘extra five minutes’ sample with weightnew2 applied, to the population proportions from step 3. If the correction was successful, the values should match.
6. Repeated for the remaining waves (2017/2018 and 2019/2020).

As this correction included treating the immigrant and ethnic minority boost respondents as a category within the boosted race variable (immigrants between 2009-2015), the method used for correction was not possible for waves prior to the existence of this sample in wave 7 (2015/2016). As such our analysis was restricted to waves 7-11 (2015-2020).

### Additional Measure Information

**Perceived Discrimination**

Participants could select they experienced the four variables in the following locations: 1) school, 2) college/university, 3) work, 4) public transport, 5) bus/train stations, 6) shopping centres/commercial places, 7) cafes, cinema/theatres, 8) pub/disco/club, 9) car parks, 10) outside, street/park, 11) home, 12) other.

**Sociodemographic Characteristics**

All variables were self-reported. Where a variable existed in continuous format, or in broader categories it was condensed into narrower categories. Monthly household income was equivalised using the OECD modified conversion scale. For each wave unique quintiles were made, before being relabelled from Category 1 (lowest) to Category 5 (highest) to produce consistent labelling across waves. Education level was categorised from participants self-reported highest education level. Self-reported current job was classified into the NS-SEC three-category version, including those not in paid employment in the last week as a fourth category.

### Additional Analyses Information

**Missing Data**

As we used a complete case analysis, we assessed whether there were differences in the characteristics of those included and not included in the analytical sample of main analysis models which contained both outcomes (perceived discrimination and probable mental health problems) and all sociodemographic characteristics as covariates (e.g. the mediation model). This included data from 2015/2016 and 2019/2020. The unweighted analytical sample in these models was 13,229 (24% missing data on 1 or more variable, including sociodemographic variables). We found that there was a significantly higher percentage of younger adults, non-White ethnic group respondents (Asian, Black, or Other), religious respondents, and respondents in the lowest SES groups (lowest income, education, and occupation) who did not have complete cases (all *p* <·001). Proportions of missingness were not different by sex and health. For our outcomes, there was no significant differences in complete cases for probable mental health problems. However, there was a slightly higher percentage of those who did not perceive discrimination who did not have complete cases compared to those who did perceive discrimination (20·5% vs. 17·8%; *p* =·001).

**Planned and Unplanned Analyses**

Primary planned analyses compared changes in perceived discrimination and probable mental health problems between the first and last available survey wave (2015/2016 vs 2019/2020). Additional planned analyses examined comparisons between pre-COVID waves (2015/2016 vs 2017/2018) and the wave immediately prior to and during COVID (2017/2018 vs 2019/2020). Repeating the primary planned analysis unweighted and exploring prior waves unweighted was unplanned. We decided to conduct this analysis to check consistency of findings after we completed the survey weight correction described above. Exploring the association between perceived discrimination and survey wave for probable mental health problems was unplanned. Remaining analyses (mediation models, using individual reasons for perceived discrimination as outcome variables, and controlling for COVID-19) were all planned.

**Correction for Multiple Comparisons**

As we had a large number of comparisons within models exploring sociodemographic differences, to reduce the false discovery rate we applied the Benjamini-Hochberg method. For our analysis, we produced Benjamini-Hochberg adjusted *p*-values. This involved ranking each *p*-value produced in order from smallest to largest, and assigning a rank number for each test (*i*). The total number of ranks or tests is *m*. We then multiplied each *p*-value by (*m*/*i*). If the adjusted *p*-value was <·05 it was determined to be significant. Where the adjusted *p*-value was greater than the result of the calculation from the larger ranked *p*-value adjustment below, the smaller adjusted *p*-value was retained (e.g. if the rank 2 calculation produced an adjusted *p*-value of ·010, but rank 1 calculation produced a value of ·015, the rank 1 adjusted *p*-value would be ·010).

**Mediation Models**

The associations between perceived discrimination and probable mental health problems (mediator and outcome) were explored using *margins* and *lincom* commands before mediations were conducted. Mediation models were conditional on finding significant changes in the same direction for both perceived discrimination and probable mental health problems between 2015/2016 and 2019/2020. Running mediation models for specific sociodemographic subgroups was conditional on subgroup difference in the changes being found for both outcomes (e.g. females having a significantly greater increase between waves in both perceived discrimination and probable mental health problems compared to the change for males). Adjustment of *p*-values is required for sociodemographic mediation models. Note that only a survey weight and cluster by primary sampling unit was applied to mediation models, as full *svy* commands are not supported with the *khb* command.

**References**

1. Wallace S, Nazroo J, Bécares L. Cumulative effect of racial discrimination on the mental health of ethnic minorities in the United Kingdom. *Am J Public Health* 2016; **106**(7): 1294–300. <http://doi.org/10.2105/ajph.2016.303121>

2. Hackett RA, Ronaldson A, Bhui K, Steptoe A, Jackson SE. Racial discrimination and health: A prospective study of ethnic minorities in the United Kingdom. *BMC Public Health* 2020; **20**(1): 1652. <http://doi.org/10.1186/s12889-020-09792-1>

3. Hackett RA, Steptoe A, Jackson SE. Sex discrimination and mental health in women: A prospective analysis. *Health Psychol* 2019; **38**(11): 1014–24. <http://doi.org/10.1037/hea0000796>

4. Hackett RA, Steptoe A, Lang RP, Jackson SE. Disability discrimination and well-being in the United Kingdom: A prospective cohort study. *BMJ Open* 2020; **10**(3): e035714. <http://doi.org/10.1136/bmjopen-2019-035714>

5. McFall S, Nandi A, Platt L. Understanding Society: UK Household Longitudinal Study: User guide to ethnicity and immigration research. 7^th^ Edition. Colchester, UK: University of Essex; 2020. <https://www.understandingsociety.ac.uk/sites/default/files/downloads/documentation/mainstage/user-guides/user-guide-ethnicity-immigration-research.pdf> (accessed June 10 2022).

6. Institute for Social and Economic Research. Understanding Society: Waves 1-11, 2009-2020 and harmonised BHPS: Waves 1-18, 1991-2009, user guide. Colchester, UK: University of Essex; 2021. <https://www.understandingsociety.ac.uk/sites/default/files/downloads/documentation/mainstage/user-guides/mainstage-user-guide.pdf> (accessed June 10 2022).

## B. Sample Characteristics and Missing Cases

**Table S1. Sample Characteristics, Perceived Discrimination and Probable Mental Health Problems by Survey Wave.**

| **Population Subgroup** | **Wave 7 (2015/2016)** | | | **Wave 9 (2017/2018)** | | | **Wave 11 (2019/2020)** | | |
| --- | --- | --- | --- | --- | --- | --- | --- | --- | --- |
|  | **Unweighted *n*** | **Unweighted %** | **Weighted**  ***n* & %** | **Unweighted *n*** | **Unweighted %** | **Weighted**  ***n* & %** | **Unweighted *n*** | **Unweighted %** | **Weighted**  ***n* & %** |
| **Sex** | **10,263** |  | **10,108** | **8359** |  | **8126** | **7130** |  | **6857** |
| Male | 4567 | 44·5% | 53·4% | 3686 | 44·1% | 52·1% | 3141 | 44·1% | 52·5% |
| Female | 5696 | 55·5% | 46·6% | 4673 | 55·9% | 47·9% | 3989 | 56·0% | 47·5% |
| **Age** | **10,262** |  | **10,107** | **8360** |  | **8127** | **7132** |  | **6859** |
| 16-34 | 3496 | 34·1% | 33·9% | 2653 | 31·7% | 32·8% | 2156 | 30·2% | 29·5% |
| 35-50 | 3672 | 35·8% | 28·1% | 2839 | 34·0% | 25·6% | 2250 | 31·6% | 23·1% |
| 51-64 | 1900 | 18·5% | 20·0% | 1741 | 20·8% | 22·7% | 1668 | 23·4% | 26·5% |
| 65+ | 1194 | 11·6% | 18·0% | 1127 | 13·5% | 18·9% | 1058 | 14·8% | 20·9% |
| **Ethnic Group** | **10,191** |  | **10,044** | **8307** |  | **8075** | **7090** |  | **6818** |
| White | 3393 | 33·3% | 84·4% | 2747 | 33·1% | 84·9% | 2353 | 33·2% | 84·0% |
| Mixed | 557 | 5·5% | 3·3% | 472 | 5·7% | 3·2% | 414 | 5·8% | 3·4% |
| Asian | 4168 | 40·9% | 7·0% | 3588 | 43·2% | 6·6% | 3131 | 44·2% | 6·6% |
| Black | 1768 | 17·4% | 3·0% | 1296 | 15·6% | 2·8% | 1016 | 14·3% | 3·1% |
| Other | 305 | 3·0% | 2·3% | 204 | 2·5% | 2·5% | 176 | 2·5% | 2·9% |
| **Religious (Identifies as belonging to a religion)** | **9897** |  | **9767** | **8235** |  | **8009** | **6990** |  | **6728** |
| Yes | 7538 | 76·2% | 51·2% | 5996 | 72·8% | 46·6% | 5057 | 72·4% | 48·3% |
| No | 2359 | 23·8% | 48·8% | 2239 | 27·2% | 53·4% | 1933 | 27·7% | 51·7% |
| **Health (Has longstanding illness/disability)** | **10,232** |  | **10,084** | **8343** |  | **8110** | **7078** |  | **6806** |
| Yes | 2662 | 26·0% | 33·3% | 2451 | 29·4% | 34·2% | 1952 | 27·6% | 34·9% |
| No | 7570 | 74·0% | 66·7% | 5892 | 70·6% | 65·8% | 5126 | 72·4% | 65·1% |
| **Equivalised Household Income per Month** | **10,148** |  | **10,000** | **8103** |  | **7879** | **6871** |  | **6599** |
| Category 1 (*lowest)* | 2029 | 20·0% | 9·6% | 1621 | 20·0% | 10·4% | 1377 | 20·0% | 9·5% |
| Category 2 | 2031 | 20·0% | 14·8% | 1621 | 20·0% | 14·8% | 1375 | 20·0% | 17·0% |
| Category 3 | 2029 | 20·0% | 18·7% | 1620 | 20·0% | 20·0% | 1375 | 20·0% | 20·0% |
| Category 4 | 2030 | 20·0% | 26·6% | 1624 | 20·0% | 24·3% | 1372 | 20·0% | 26·5% |
| Category 5 *(highest)* | 2029 | 20·0% | 30·2% | 1617 | 20·0% | 30·4% | 1372 | 20·0% | 27·0% |
| **Education Level** | **9355** |  | **9206** | **7800** |  | **7570** | **6771** |  | **6499** |
| Other/No Qualification | 1704 | 18·2% | 14·9% | 1372 | 17·6% | 13·7% | 1029 | 15·2% | 11·9% |
| High School Qualification | 3525 | 37·7% | 40·0% | 2905 | 37·2% | 39·7% | 2512 | 37·1% | 40·2% |
| University Degree | 4126 | 44·1% | 45·1% | 3523 | 45·2% | 46·5% | 3230 | 47·7% | 47·9% |
| **Current Job** | **10,152** |  | **10,005** | **8121** |  | **7896** | **6841** |  | **6575** |
| Not in paid employment in the last week | 4240 | 41·8% | 37·2% | 3536 | 43·5% | 38·7% | 3167 | 46·3% | 41·6% |
| Routine | 2154 | 21·2% | 20·4% | 1617 | 19·9% | 20·1% | 1169 | 17·1% | 18·1% |
| Intermediate | 1414 | 13·9% | 13·9% | 1085 | 13·4% | 14·2% | 911 | 13·3% | 12·8% |
| Management & Professional | 2344 | 23·1% | 28·5% | 1883 | 23·2% | 27·0% | 1594 | 23·3% | 27·5% |
| **Perceived Discrimination** | **9492** |  | **9371** | **8051** |  | **7829** | **7025** |  | **6757** |
| Yes | 1784 | 18·8% | 14·6% | 1722 | 21·4% | 18·2% | 1578 | 22·5% | 20·1% |
| No | 7708 | 81·2% | 85·4% | 6329 | 78·6% | 81·8% | 5447 | 77·5% | 79·9% |
| ***Due to:^a^*** | **1784** |  | **1681** | **1722** |  | **1606** | **1578** |  | **1400** |
| Sex | 554 | 31·1% | 40·0% | 588 | 34·2% | 44·6% | 570 | 36·1% | 44·2% |
| Age | 314 | 17·6% | 32·7% | 377 | 21·9% | 41·2% | 438 | 27·8% | 39·7% |
| Ethnicity-based (ethnicity, nationality, or language/accent) | 860 | 48·2% | 28·0% | 851 | 49·4% | 26·2% | 771 | 48·9% | 24·6% |
| Sexual Orientation | 64 | 3·6% | 3·3% | 61 | 3·5% | 3·7% | 65 | 4·1% | 3·9% |
| Health/Disability | 183 | 10·3% | 13·7% | 201 | 11·7% | 17·2% | 318 | 20·2% | 23·0% |
| Religion | 623 | 34·9% | 11·7% | 626 | 36·4% | 12·5% | 442 | 28·0% | 8·4% |
| Dress/Appearance | 364 | 20·4% | 22·3% | 445 | 25·8% | 33·4% | 398 | 25·2% | 27·6% |
| **Probable Mental Health Problems** | **9340** |  | **9196** | **7427** |  | **7206** | **6654** |  | **6377** |
| Yes | 1724 | 18·5% | 17·9% | 1368 | 18·4% | 17·7% | 1468 | 22·1% | 22·1% |
| No | 7616 | 81·5% | 82·1% | 6059 | 81·6% | 82·3% | 5186 | 77·9% | 77·9% |

Note: Overall ‘extra five minutes’ unweighted sample numbers for wave 7 (2015/2016) is 10,264, for wave 9 (2017/2018) is 8360, and for wave 11 (2019/2020) is 7132.

^a^These percentages refer to the percentage allocating this reason from the proportion who have selected ‘yes’ to the discrimination experience, as opposed to the full sample.

**Table S2. Missing Cases by Variable and Survey Wave.**

| **Population Subgroup** | **Wave 7 (2015/2016)** | | | **Wave 9 (2017/2018)** | | | **Wave 11 (2019/2020)** | | |
| --- | --- | --- | --- | --- | --- | --- | --- | --- | --- |
|  | **Unweighted *n*** | **Missing *n*** | **Missing %** | **Unweighted *n*** | **Missing *n*** | **Missing %** | **Unweighted *n*** | **Missing *n*** | **Missing %** |
| Sex | 10,263 | 1 | 0·01% | 8359 | 1 | 0·01% | 7130 | 2 | 0·03% |
| Age | 10,262 | 2 | 0·02% | 8360 | 0 | 0·0% | 7132 | 0 | 0·0% |
| Ethnic Group | 10,191 | 73 | 0·7% | 8307 | 53 | 0·6% | 7090 | 42 | 0·6% |
| Religious (Identifies as belonging to a religion) | 9897 | 367 | 3·6% | 8235 | 125 | 1·5% | 6990 | 142 | 2·0% |
| Health (Has longstanding illness/disability) | 10,232 | 32 | 0·3% | 8343 | 17 | 0·2% | 7078 | 54 | 0·8% |
| Equivalised Household Income per Month | 10,148 | 116 | 1·1% | 8103 | 257 | 3·1% | 6871 | 261 | 3·7% |
| Education Level | 9355 | 909 | 8·9% | 7800 | 560 | 6·7% | 6771 | 361 | 5·1% |
| Current Job | 10,152 | 112 | 1·1% | 8121 | 239 | 2·9% | 6841 | 291 | 4·1% |
| Perceived Discrimination | 9492 | 772 | 7·5% | 8051 | 309 | 3·7% | 7025 | 107 | 1·5% |
| Probable Mental Health Problems | 9340 | 924 | 9·0% | 7427 | 933 | 11·2% | 6654 | 478 | 6·7% |

Note: Overall ‘extra five minutes’ unweighted sample numbers for wave 7 (2015/2016) is 10,264, for wave 9 (2017/2018) is 8360, and for wave 11 (2019/2020) is 7132.

## C. Results of Primary Analyses Using Unweighted Analysis


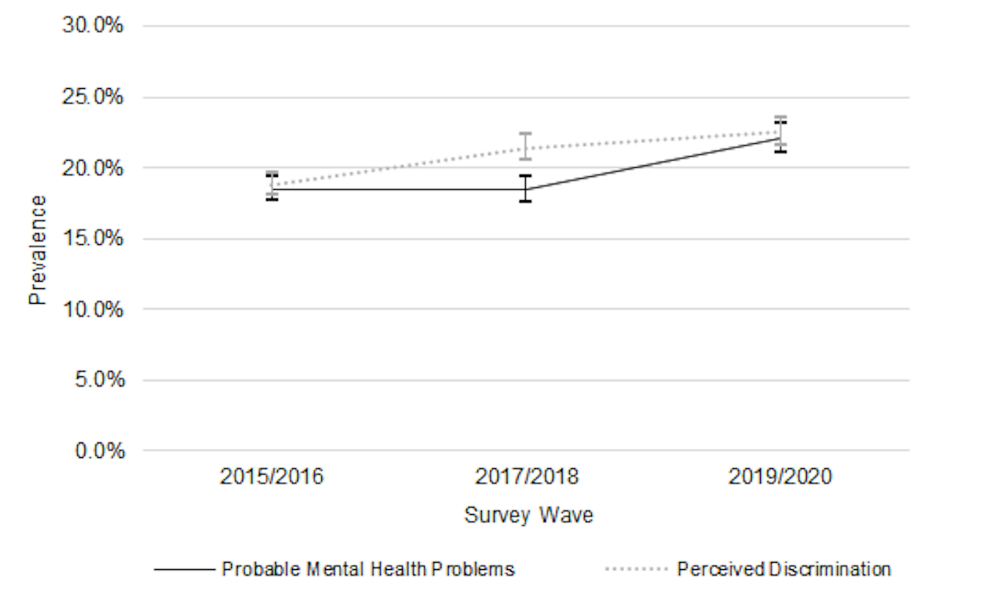


***

**Figure S1. Unweighted Prevalence of Perceived Discrimination and Probable Mental Health Problems by Survey Waves Included in Main Analysis.**

The graph shows unweighted descriptive prevalence percentages and confidence intervals. In models which controlled for all sociodemographic characteristics, the changes in both outcomes between 2015/2016 and 2019/2020 were significant. *** *p* <·001.

Weighted models from the main analysis (2015/2016 vs 2019/2020) were repeated unweighted, with standard errors clustered by the individual identifier to account for the repeated cross-sectional design.

**Changes in Perceived Discrimination**

Consistent with the weighted analysis, between 2015/2016 to 2019/2020 perceived discrimination increased significantly by 3·5% between waves (95% CI: 2·3-4·8, *p* <·001).

**Changes in Probable Mental Health Problems**

Consistent with the weighed analysis, between 2015/2016 to 2019/2020 probable mental health problems increased significantly by 3·2% between waves (95% CI: 2·0-4·5, *p* <·001).

**Differences between Sociodemographic Groups in the Changes in Perceived Discrimination and Probable Mental Health Problems**

Unlike the weighted analysis, there was statistical evidence that survey wave interacted with some of the sociodemographic variables for perceived discrimination and probable mental health problems (see Table S3), indicating that unweighted changes from 2015/2016 to 2019/2020 were different across some sociodemographic groups.

**Table S3. Unweighted Differences between Sociodemographic Groups in the Changes in Perceived Discrimination and Probable Mental Health Problems from 2015/2016 to 2019/2020.**

| **Population Subgroup** | **Difference between Subgroups in the Changes in Perceived Discrimination from 2015/2016 to 2019/2020^a^** | | **Difference between Subgroups in the Changes in Probable Mental Health Problems from 2015/2016 to 2019/2020^b^** | |
| --- | --- | --- | --- | --- |
|  | **%** | **(95% CI)** | **%** | **(95% CI)** |
| **Sex (Comparison: Males)** |  | |  | |
| Females | +1·6 | (-0·9 to 4·1) | +2·2 | (-0·3 to 4·6) |
| **Age Group (Comparison: 16-34 Years)** |  | |  | |
| 35-50 Years | -1·9 | (-5·2 to 1·4) | -0·7 | (-4·1 to 2·7) |
| 51-64 Years | -0·6 | (-4·2 to 2·9) | -1·6 | (-5·2 to 1·9) |
| 65+ Years | +0·7 | (-3·2 to 4·7) | -3·1 | (-6·5 to 0·4) |
| **Ethnic Group (Comparison: White Respondents)** |  | |  | |
| Mixed | +2·3 | (-3·5 to 8·1) | +0·7 | (-4·8 to 6·2) |
| Asian | -4·8^**^ | (-7·7 to -2·0) | -3·0 | (-5·8 to -0·1) |
| Black | +0·5 | (-3·2 to 4·3) | -5·0^*^ | (-8·6 to -1·4) |
| Other | -1·6 | (-9·7 to 6·5) | +1·3 | (-7·5 to 10·2) |
| **Religious (Comparison: Does not identify as belonging to a religion)** |  | |  | |
| Identifies as belonging to a religion | -4·7^**^ | (-7·7 to -1·8) | -2·9 | (-5·7 to 0·02) |
| **Health (Comparison: Does not have a longstanding illness/disability)** |  | |  | |
| Has a longstanding illness/disability | +3·6^*^ | (0·5-6·8) | -0·6 | (-3·9 to 2·6) |
| **Equivalised Household Income Per Month (Comparison: Category 1)** |  | |  | |
| Category 2 | +3·1 | (-1·1 to 7·3) | +1·0 | (-3·3 to 5·2) |
| Category 3 | +3·5 | (-0·8 to 7·8) | +0·7 | (-3·5 to 4·9) |
| Category 4 | +5·5^*^ | (1·4-9·7) | +4·2 | (0·05-8·4) |
| Category 5 | +6·7^**^ | (2·6-10·8) | +2·0 | (-2·1 to 6·1) |
| **Education Level (Comparison: Other/No Qualification)** |  | |  | |
| High School Qualification | +0·8 | (-2·8 to 4·4) | +1·5 | (-2·2 to 5·1) |
| University Degree | +4·0^*^ | (0·5-7·5) | +4·0 | (0·5-7·5) |
| **Current Job (Comparison: Not in Paid Employment in the Last Week)** |  | |  | |
| Routine | -4·1^*^ | (-7·6 to -0·6) | +1·8 | (-1·7 to 5·2) |
| Intermediate | -2·4 | (-6·3 to 1·6) | +3·2 | (-0·7 to 7·1) |
| Management & Professional | +1·1 | (-2·2 to 4·3) | +2·0 | (-1·3 to 5·3) |

Note: Interaction terms were added to multivariable models containing all sociodemographic characteristics as covariates. *P*-values are adjusted using the Benjamini-Hochberg method. Differences displayed are lincom differences between the changes in predicted probabilities between waves (marginal effects), multiplied by 100 to get percentage point differences. The difference in the changes are between the displayed comparison group and non-comparison group (e.g. males vs. females). Negative values indicate that the change for the comparison group was greater than the change for the non-comparison group.

^a^Perceived discrimination unweighted *n* = 14,281.

^b^Probable mental health problems unweighted *n* = 13,731.

^*^ *p* <·05; ^**^ *p* <·01; ^***^ *p* <·001.

**Changes by Population Subgroup**

For details of the specific changes for each subgroup, see Table S4. Unweighted changes in perceived discrimination between 2015/2016 to 2019/2020 were consistent with the weighted analysis. Exploring unweighted changes in probable mental health problems between waves revealed a larger number of subgroups with a significant increase between waves, compared to the weighted analysis.

**Table S4. Unweighted Percentage Point Changes in Perceived Discrimination and Probable Mental Health Problems Between 2015/2016 and 2019/2020, by Population Subgroup.**

| **Population Subgroup** | **Change between 2015/2016 – 2019/2020^a^** | | | |
| --- | --- | --- | --- | --- |
|  | **Perceived Discrimination^b^** | | **Probable Mental Health Problems^c^** | |
|  | **%** | **(95% CI)** | **%** | **(95% CI)** |
| **Overall** | +3·5^***^ | (2·3-4·8) | +3·2^***^ | (2·0-4·5) |
| **By Sex** |  | |  | |
| Males | +2·7^**^ | (0·9-4·4) | +2·0 | (0·2-3·8) |
| Females | +4·2^***^ | (2·5-6·0) | +4·2^***^ | (2·5-5·9) |
| **By Age** |  | |  | |
| 16-34 | +4·2^**^ | (1·7-6·7) | +4·3^**^ | (1·7-6·8) |
| 35-50 | +2·3 | (0·1-4·5) | +3·6^**^ | (1·4-5·8) |
| 51-64 | +3·6^*^ | (1·0-6·1) | +2·7 | (0·2-5·2) |
| 65+ | +4·9^**^ | (1·9-8·0) | +1·2 | (-1·2 to 3·6) |
| **By Ethnic Group** |  | |  | |
| White | +5·4^***^ | (3·3-7·5) | +5·2^***^ | (3·1-7·2) |
| Mixed | +7·7^*^ | (2·3-13·1) | +5·9 | (0·8-11·0) |
| Asian | +0·6 | (-1·4 to 2·6) | +2·2 | (0·2-4·2) |
| Black | +5·9^**^ | (2·8-9·1) | +0·2 | (-2·9 to 3·2) |
| Other | +3·8 | (-4·0 to 11·7) | +6·5 | (-2·2 to 15·1) |
| **By Religious** |  | |  | |
| Identifies as belonging to a religion | +2·4^**^ | (0·8-3·8) | +2·5^**^ | (1·0-3·9) |
| Does not identify as belonging to a religion | +7·1^***^ | (4·6-9·6) | +5·3^***^ | (2·8-7·8) |
| **By Health** |  | |  | |
| Has a longstanding illness/disability | +6·2^***^ | (3·4-9·0) | +2·8 | (-0·1 to 5·6) |
| Does not have a longstanding illness/disability | +2·6^**^ | (1·2-4·0) | +3·4^***^ | (2·0-4·7) |
| **By Equivalised Household Income Per Month** |  | |  | |
| Category 1 (lowest) | -0·2 | (-3·2 to 2·8) | +1·7 | (-1·3 to 4·6) |
| Category 2 | +2·9 | (-0·04 to 5·8) | +2·6 | (-0·3 to 5·6) |
| Category 3 | +3·3 | (0·3-6·3) | +2·3 | (-0·6 to 5·2) |
| Category 4 | +5·3^**^ | (2·5-8·2) | +5·9^**^ | (3·0-8·8) |
| Category 5 (highest) | +6·5^***^ | (3·7-9·3) | +3·7^*^ | (0·8-6·5) |
| **By Education Level** |  | |  | |
| Other/No Qualification | +1·4 | (-1·5 to 4·4) | +0·8 | (-2·1 to 3·8) |
| High School Qualification | +2·2 | (0·1-4·3) | +2·3 | (0·3 to 4·3) |
| University Degree | +5·4^***^ | (3·6-7·3) | +4·8^***^ | (3·0 to 6·7) |
| **By Current Job** |  | |  | |
| Not in paid employment in the last week | +4·4^***^ | (2·4-6·3) | +2·0 | (0·02-4·0) |
| Routine | +0·3 | (-2·6 to 3·2) | +3·8^*^ | (0·9-6·6) |
| Intermediate | +2·0 | (-1·5 to 5·5) | +5·2^*^ | (1·8-8·6) |
| Management & Professional | +5·5^***^ | (2·9-8·1) | +4·0^*^ | (1·3-6·6) |

Note: ^a^Change between 2015/2016 – 2019/2020 is the change in the predicted probability of perceiving discrimination or experiencing probable mental health problems. Interaction terms were added to multivariable models containing all sociodemographic characteristics as covariates. Differences displayed are lincom differences in predicted probabilities (marginal effects), multiplied by 100 for percentage point differences. *P*-values are adjusted using the Benjamini-Hochberg method.

^b^Perceived discrimination unweighted *n* = 14,281.

^c^Probable mental health problems unweighted *n* = 13,731.

^*^ *p* <·05; ^**^ *p* <·01; ^***^ *p* <·001.

**Mediation Analyses**

Consistent with the weighted analysis, we found that the change in probable mental health problems observed between 2015/2016 to 2019/2020 was in part mediated (10·5% of association) by the increase in perceived discrimination observed during the same time period (see Table S5).

**Table S5. Unweighted Mediation Models Exploring Perceived Discrimination Experiences as a Mediator of the Association Between Wave and Probable Mental Health Problems.**

| **IV** | **Mental Health** | | **Effect Ratio** |
| --- | --- | --- | --- |
|  | **B** | **95% CIs** |  |
| **Wave (Reference: Wave 7 [2015/2016])** |  |  |  |
| Total effect of Wave (Wave 11 [2019/2020]) | 0·21^***^ | 0·12 to 0·29 |  |
| Direct effect of Wave (Wave 11 [2019/2020]) | 0·19^***^ | 0·10 to 0·27 |  |
| *Indirect effect via perceived discrimination* | *0·02^***^* | *0·01 to 0·03* | *10·5%* |

Note: *n* = 13,229. Model controls for all sociodemographic characteristics (adjusted model).

^*^*p* <·05; ^**^*p* <·01; ^***^*p* <·001.

## D. Repeated Analyses with Other Wave Comparisons (2015/2016 vs 2017/2018; 2017/2018 vs 2019/2020)

**Table S6.** **Differences between Sociodemographic Groups in the Changes in Perceived Discrimination and Probable Mental Health Problems from 2015/2016 to 2017/2018 and from 2017/2018 to 2019/2020.**

| **Population Subgroup** | **Difference between Subgroups in the Changes in Perceived Discrimination from 2015/2016 to 2017/2018^a^** | | **Difference between Subgroups in the Changes in Probable Mental Health Problems from 2015/2016 to 2017/2018^b^** | | **Difference between Subgroups in the Changes in Perceived Discrimination from 2017/2018 to 2019/2020^c^** | | **Difference between Subgroups in the Changes in Probable Mental Health Problems from 2017/2018 to 2019/2020^d^** | |
| --- | --- | --- | --- | --- | --- | --- | --- | --- |
|  | **%** | **(95% CI)** | **%** | **(95% CI)** | **%** | **(95% CI)** | **%** | **(95% CI)** |
| **Sex (Comparison: Males)^e^** |  | |  | |  | |  | |
| Females | +1·7 | (-4·0 to 7·4) | +4·8 | (-1·4 to 11·0) | -0·3 | (-5·7 to 5·2) | -0·8 | (-6·4 to 4·9) |
| **Age Group (Comparison: 16-34 Years)^f^** |  | |  | |  | |  | |
| 35-50 Years | -9·3 | (-16·8 to -1·8) | +6·7 | (-3·5 to 16·9) | +4·7 | (-3·3 to 12·7) | -1·1 | (-10·6 to 8·4) |
| 51-64 Years | -6·5 | (-14·1 to 1·1) | +3·6 | (-5·1 to 12·2) | +11·7 | (3·3-20·1) | -0·2 | (-8·2 to 7·8) |
| 65+ Years | -8·3 | (-15·5 to -1·0) | +3·4 | (-3·8 to 10·6) | +6·9 | (-1·0 to 14·7) | -1·5 | (-9·1 to 6·1) |
| **Ethnic Group (Comparison: White Respondents)^g^** |  | |  | |  | |  | |
| Mixed | -14·6 | (-27·7 to -1·5) | +4·7 | (-6·5 to 15·8) | +12·2 | (1·3-23·1) | -5·1 | (-13·5 to 3·3) |
| Asian | -4·5 | (-12·2 to 3·1) | -2·4 | (-9·0 to 4·2) | +5·7 | (-3·9 to 15·2) | -3·1 | (-10·7 to 4·6) |
| Black | -4·8 | (-16·6 to 7·0) | -4·9 | (-13·4 to 3·6) | -6·2 | (-16·9 to 4·4) | -3·8 | (-13·8 to 6·2) |
| Other | +19·5 | (-5·9 to 45·0) | +12·9 | (-8·2 to 33·9) | -31·1 | (-58·4 to -3·7) | -22·4 | (-46·0 to 1·1) |
| **Religious (Comparison: Does not identify as belonging to a religion)^h^** |  | |  | |  | |  | |
| Identifies as belonging to a religion | -4·1 | (-9·3 to 1·1) | -1·6 | (-7·7 to 4·4) | +0·9 | (-4·8 to 6·7) | -1·2 | (-7·2 to 4·8) |
| **Health (Comparison: Does not have a longstanding illness/disability)^i^** |  | |  | |  | |  | |
| Has a longstanding illness/disability | 0·0 | (-5·9 to 5·9) | +5·2 | (-1·4 to 11·9) | +5·2 | (-2·6 to 13·0) | -5·2 | (-11·9 to 1·5) |
| **Equivalised Household Income Per Month (Comparison: Category 1)^j^** |  | |  | |  | |  | |
| Category 2 | -1·6 | (-11·4 to 8·2) | -4·4 | (-17·2 to 8·5) | -10·7 | (-23·9 to 2·6) | +3·5 | (-10·6 to 17·5) |
| Category 3 | -1·3 | (-11·5 to 9·0) | -8·8 | (-20·9 to 3·3) | -2·9 | (-16·1 to 10·4) | +7·2 | (-5·0 to 19·5) |
| Category 4 | +4·1 | (-4·7 to 13·0) | -2·5 | (-13·3 to 8·3) | -1·4 | (-12·8 to 9·9) | +7·5 | (-3·5 to 18·6) |
| Category 5 | -0·2 | (-8·9 to 8·5) | -9·4 | (-20·2 to 1·3) | +0·6 | (-10·2 to 11·3) | +10·3 | (-0·9 to 21·4) |
| **Education Level (Comparison: Other/No Qualification)^k^** |  | |  | |  | |  | |
| High School Qualification | +2·7 | (-5·3 to 10·7) | -7·0 | (-15·5 to 1·4) | +4·0 | (-6·0 to 14·0) | +8·8 | (-0·8 to 18·4) |
| University Degree | +2·8 | (-5·5 to 11·2) | -4·7 | (-14·3 to 4·9) | +4·3 | (-5·2 to 13·7) | +9·7 | (-0·1 to 19·5) |
| **Current Job (Comparison: Not in Paid Employment in the Last Week)^l^** |  | |  | |  | |  | |
| Routine | +0·4 | (-7·9 to 8·6) | +1·8 | (-6·1 to 9·7) | -0·09 | (-10·2 to 10·0) | -2·0 | (-10·4 to 6·4) |
| Intermediate | +4·0 | (-5·3 to 13·4) | +3·8 | (-4·5 to 12·1) | -6·6 | (-15·5 to 2·3) | -4·4 | (-13·0 to 4·3) |
| Management & Professional | -2·9 | (-9·2 to 3·3) | +1·4 | (-6·1 to 8·9) | +7·4 | (0·4-14·5) | +6·5 | (-1·8 to 14·8) |

Note: Interaction terms were added to multivariable models containing all sociodemographic characteristics as covariates. *P*-values are adjusted using the Benjamini-Hochberg method. Differences displayed are lincom differences between the changes in predicted probabilities between waves (marginal effects), multiplied by 100 to get percentage point differences. The difference in the changes are between the displayed comparison group and non-comparison group (e.g. males vs. females). Negative values indicate that the change for the comparison group was greater than the change for the non-comparison group. For details of the changes in the outcomes between waves by subgroup, see Table S7.

^a^Weighted *n* = 15,107.

^b^Weighted *n* = 14,474.

^c^Weighted *n* = 12,595.

^d^Weighted *n* = 12,014.

**Association Between Perceived Discrimination and Probable Mental Health Problems**

For the 2015/2016 vs 2017/2018 sample, probable mental health problems were significantly greater by 10·6% (95% CI: 5·4-15·8, *p* <·001) for those who had perceived discrimination, compared to those who had not. For the 2017/2018 vs 2019/2020 sample, probable mental health problems were significantly greater by 15·0% (95% CI: 10·0-20·0, *p* <·001) for those who had perceived discrimination, compared to those who had not.

We explored an interaction between perceived discrimination and survey wave (2015/2016 vs 2017/2018) for the prediction of probable mental health problems in a model that controlled for all sociodemographic characteristics. This revealed that the size of the change in probable mental health problems between 2015/2016 and 2017/2018 was not meaningfully different for those who did and did not perceive discrimination (difference in the change = +6·7%, 95% CI: -1·4 to 14·8, *p* =·106). Specifically, those who had perceived discrimination had a 4.0% non-significant increase in probable mental health problems between 2015/2016 to 2017/2018 (95% CI: -2·7 to 10·7, *p* =·244), and those who had not perceived discrimination had a non-significant 2.7% decrease between waves (95% CI: -6·3 to 0·9, *p* =·140).

We also explored an interaction between perceived discrimination and survey wave (2017/2018 vs 2019/2020) for the prediction of probable mental health problems in a model that controlled for all sociodemographic characteristics. This revealed that the size of the change in probable mental health problems between 2017/2018 and 2019/2020 was not meaningfully different for those who did and did not perceive discrimination (difference in the change = -1·4%, 95% CI: -10·8 to 7·9, *p* =·762). Specifically, those who had perceived discrimination had a 3.8% non-significant increase in probable mental health problems between 2017/2018 to 2019/2020 (95% CI: -4·6 to 12·2, *p* =·374), and those who had not perceived discrimination had a significant 5.3% increase between waves (95% CI: 2·1-8·5, *p* =·001).

Note exploratory mediation models were not conducted, as perceived discrimination experiences did not significantly change over time or the survey wave and mental health association (total effect) was not significant.

## E. Population Subgroup Changes Between Waves

To allow us to explore subgroup differences within changes in perceived discrimination and probable mental health problems between waves (whether there was an interaction effect) we firstly looked at changes in outcomes by subgroup. Results in Table S7 can be used to understand the difference values within Table 2 and Table S6.

**Association Between Perceived Discrimination and Probable Mental Health Problems**

We explored an interaction between perceived discrimination and survey wave (2015/2016 vs 2019/2020) for the prediction of probable mental health problems in a model that controlled for all sociodemographic characteristics. This revealed that the size of the change in probable mental health problems between 2015/2016 and 2019/2020 was not meaningfully different for those who did and did not perceive discrimination (difference in the change = +5·7%, 95% CI: -3·0 to 14·3, *p* =·198). Specifically, those who had perceived discrimination had an 8·3% significant increase in probable mental health problems between 2015/2016 to 2019/2020 (95% CI: 0·7-15·8, *p* =·031), and those who had not perceived discrimination had a non-significant 2.6% increase between waves (95% CI: -1·1 to 6·3, *p* =·171).

**Table S7. Percentage Point Changes in Perceived Discrimination and Probable Mental Health Problems Between 2015/2016 to 2019/2020 (Main Analysis), 2015/2016 to 2017/2018, and 2017/2018 to 2019/2020, by Population Subgroup.**

| **Population Subgroup** | **Perceived Discrimination** | | | | | | **Probable Mental Health Problems** | | | | | |
| --- | --- | --- | --- | --- | --- | --- | --- | --- | --- | --- | --- | --- |
|  | **Change between 2015/2016 – 2017/2018^a^** | | **Change between 2017/2018 – 2019/2020^b^** | | **Change between 2015/2016 – 2019/2020^c^** | | **Change between 2015/2016 – 2017/2018^a^** | | **Change between 2017/2018 – 2019/2020^b^** | | **Change between 2015/2016 – 2019/2020^c^** | |
|  | **%** | **(95% CI)** | **%** | **(95% CI)** | **%** | **(95% CI)** | **%** | **(95% CI)** | **%** | **(95% CI)** | **%** | **(95% CI)** |
| **Overall** | +4·0^**^ | (1·3-6·6) | +2·3 | (-0·6 to 5·2) | +6·1^***^ | (3·4-8·8) | -0·9 | (-3·8 to 2·0) | +5·3^***^ | (2·4-8·2) | +4·5^**^ | (1·3-7·7) |
| **By Sex** |  | |  | |  |  |  |  |  |  |  |  |
| Males | +3·1 | (-0·05 to 6·3) | +2·4 | (-1·3 to 6·2) | +5·3^*^ | (1·9-8·7) | -3·3 | (-7·6 to 1·1) | +5·7^*^ | (2·2-9·2) | +2·6 | (-2·0 to 7·2) |
| Females | +4·9 | (0·3-9·4) | +2·2 | (-2·0 to 6·4) | +7·0^*^ | (2·6-11·4) | +1·6 | (-2·6 to 5·7) | +4·9 | (0·4-9·4) | +6·5 | (2·1-11·0) |
| **By Age** |  | |  | |  |  |  |  |  |  |  |  |
| 16-34 | +9·6^*^ | (3·6-15·5) | -3·3 | (-9·6 to 2·9) | +6·2 | (0·4-12·0) | -4·1 | (-10·3 to 2·1) | +5·9 | (-0·2 to 11·9) | +1·9 | (-5·5 to 9·3) |
| 35-50 | +0·3 | (-4·6 to 5·2) | +1·4 | (-4·5 to 7·3) | +2·1 | (-2·3 to 6·5) | +2·6 | (-5·0 to 10·2) | +4·8 | (-2·5 to 12·0) | +7·7 | (-0·2 to 15·6) |
| 51-64 | +3·1 | (-1·7 to 7·9) | +8·4 | (2·9-13·9) | +11·1^**^ | (5·6-16·7) | -0·6 | (-6·4 to 5·2) | +5·6 | (0·6-10·7) | +5·0 | (-0·4 to 10·5) |
| 65+ | +1·3 | (-2·8 to 5·5) | +3·5 | (-1·0 to 8·1) | +4·9 | (-0·3 to 10·0) | -0·8 | (-3·8 to 2·3) | +4·4 | (0·06-8·7) | +3·0 | (-1·5 to 7·6) |
| **By Ethnic Group** |  | |  | |  |  |  |  |  |  |  |  |
| White | +4·6^*^ | (1·7-7·4) | +2·5 | (-0·6 to 5·7) | +6·7^***^ | (3·7-9·8) | -1·1 | (-4·3 to 2·2) | +6·4^**^ | (3·2-9·7) | +5·4 | (1·8-9·0) |
| Mixed | -10·0 | (-22·7 to 2·7) | +14·7 | (4·2-25·3) | +4·7 | (-12·0 to 21·4) | +3·6 | (-7·4 to 14·6) | +1·3 | (-6·4 to 9·1) | +6·2 | (-7·4 to 19·8) |
| Asian | 0·04 | (-7·1 to 7·2) | +8·2 | (-0·8 to 17·3) | +8·3 | (0·8-15·7) | -3·5 | (-9·4 to 2·4) | +3·3 | (-3·6 to 10·3) | -0·1 | (-7·1 to 6·9) |
| Black | -0·2 | (-11·7 to 11·2) | -3·7 | (-13·8 to 6·4) | -3·6 | (-15·5 to 8·3) | -6·0 | (-13·8 to 1·8) | +2·6 | (-6·7 to 12·0) | -3·7 | (-12·9 to 5·4) |
| Other | +24·1 | (-13·7 to 49·6) | -28·5 | (-55·8 to -1·3) | -4·3 | (-19·5 to 11·0) | +11·8 | (-8·9 to 32·4) | -16·0 | (-39·3 to 7·3) | -4·2 | (-31·0 to 22·7) |
| **By Religious** |  | |  | |  |  |  |  |  |  |  |  |
| Identifies as belonging to a religion | +1·9 | (-1·4 to 5·2) | +2·8 | (-1·1 to 6·7) | +4·4 | (0·6-8·2) | -1·7 | (-5·7 to 2·2) | +4·7 | (0·8-8·6) | +2·8 | (-1·7 to 7·3) |
| Does not identify as belonging to a religion | +6·0 | (2·0-10·0) | +1·9 | (-2·4 to 6·1) | +7·8^**^ | (3·6-12·0) | -0·1 | (-4·5 to 4·3) | +5·9 | (1·5-10·2) | +6·1 | (1·1-11·1) |
| **By Health** |  | |  | |  |  |  |  |  |  |  |  |
| Has a longstanding illness/disability | +3·9 | (-1·3 to 9·2) | +5·7 | (-0·6 to 12·0) | +9·3^*^ | (3·2-15·4) | +2·7 | (-3·0 to 8·3) | +1·9 | (-3·4 to 7·2) | +4·7 | (-1·5 to 11·0) |
| Does not have a longstanding illness/disability | +3·9 | (1·0-6·8) | +0·5 | (-3·1 to 4·1) | +4·3^*^ | (1·0-7·6) | -2·6 | (-5·9 to 0·8) | +7·1^**^ | (3·5-10·7) | +4·3 | (0·6-7·9) |
| **By Equivalised Household Income Per Month** |  | |  | |  |  |  |  |  |  |  |  |
| Category 1 (lowest) | +3·5 | (-2·9 to 9·9) | +5·0 | (-4·0 to 14·0) | +8·5 | (0·2-16·9) | +4·9 | (-4·2 to 13·9) | -1·4 | (-10·8 to 7·9) | +4·0 | (-4·1 to 12·1) |
| Category 2 | +1·9 | (-4·9 to 8·7) | -5·7 | (-14·2 to 2·7) | -3·1 | (-9·1 to 2·9) | +0·5 | (-7·5 to 8·5) | +2·0 | (-7·0 to 11·0) | +2·4 | (-5·9 to 10·7) |
| Category 3 | +2·3 | (-5·0 to 9·5) | +2·1 | (-7·1 to 11·3) | +3·4 | (-4·7 to 11·5) | -4·0 | (-10·9 to 3·0) | +5·8 | (-1·4 to 13·0) | +1·0 | (-6·5 to 8·5) |
| Category 4 | +7·7 | (1·8-13·5) | +3·5 | (-2·7 to 9·8) | +10·7^**^ | (5·4-16·1) | +2·4 | (-3·1 to 7·9) | +6·1 | (0·1-12·1) | +8·5 | (2·1 to 14·9) |
| Category 5 (highest) | +3·3 | (-2·4 to 9·0) | +5·5 | (0·09-11·0) | +8·8^*^ | (3·2-14·5) | -4·6 | (-11·0 to 1·9) | +8·8 | (2·1-15·6) | +4·5 | (-1·3 to 10·2) |
| **By Education Level** |  | |  | |  |  |  |  |  |  |  |  |
| Other/No Qualification | +1·6 | (-5·5 to 8·7) | -1·3 | (-9·8 to 7·3) | +0·2 | (-7·8 to 8·2) | +4·1 | (-3·5 to 11·7) | -2·8 | (-11·1 to 5·5) | +1·0 | (-6·0 to 8·0) |
| High School Qualification | +4·3 | (0·1-8·5) | +2·7 | (-2·0 to 7·4) | +6·9^*^ | (2·6-11·2) | -2·9 | (-6·7 to 0·8) | +6·0 | (1·4-10·5) | +2·8 | (-2·0 to 7·6) |
| University Degree | +4·4 | (0·3-8·6) | +3·0 | (-1·7 to 7·6) | +7·2^**^ | (3·1-11·2) | -0·6 | (-5·6 to 4·5) | +6·9^*^ | (2·2-11·5) | +6·9 | (2·0-11·8) |
| **By Current Job** |  | |  | |  |  |  |  |  |  |  |  |
| Not in paid employment in the last week | +4·1 | (-0·2 to 8·3) | +1·2 | (-3·8 to 6·3) | +5·0 | (0·4-9·6) | -2·3 | (-6·8 to 2·3) | +4·5 | (-0·6 to 9·6) | +2·0 | (-3·5 to 7·5) |
| Routine | +4·4 | (-2·5 to 11·4) | +1·1 | (-6·4 to 8·7) | +5·4 | (-14·6 to 12·3) | -0·4 | (-6·4 to 5·6) | +2·5 | (-3·6 to 8·5) | +2·2 | (-5·9 to 10·2) |
| Intermediate | +8·1 | (-0·01 to 16·2) | -5·4 | (-12·8 to 2·1) | +2·9 | (-3·6 to 9·5) | +1·5 | (-5·7 to 8·8) | +0·1 | (-6·9 to 7·1) | +2·4 | (-3·8 to 8·7) |
| Management & Professional | +1·2 | (-3·6 to 5·9) | +8·7 | (3·3-14·1) | +9·8^**^ | (4·6-15·0) | -0·8 | (-7·0 to 5·3) | 11·0^*^ | (4·6-17·4) | +10·1^*^ | (4·2-16·0) |

Note: Change between waves is the change in the predicted probability of perceiving discrimination or experiencing probable mental health problems. Interaction terms were added to multivariable models containing all sociodemographic characteristics as covariates. Differences displayed are lincom differences in predicted probabilities (marginal effects), multiplied by 100 for percentage point differences. *P*-values are adjusted using the Benjamini-Hochberg method.

^a^For change between 2015/2016 and 2017/2018: Perceived discrimination weighted *n* = 15,107. Probable mental health problems weighted *n* = 14,474.

^b^For change between 2017/2018 and 2019/2020: Perceived discrimination weighted *n* = 12,595. Probable mental health problems weighted *n* = 12,014.

^c^Main analysis waves. For change between 2015/2016 and 2019/2020: Perceived discrimination weighted *n* = 14,172. Probable mental health problems weighted *n* = 13,713.

^*^ *p* <·05; ^**^ *p* <·01; ^***^ *p* <·001.

## F. Unadjusted Mediation Models

Consistent with the adjusted analysis, we found that the change in probable mental health problems observed between 2015/2016 to 2019/2020 was in part mediated (20·5% of association) by the increase in perceived discrimination observed during the same time period (see Table S8).

**Table S8. Unadjusted Mediation Models Exploring Perceived Discrimination Experiences as a Mediator of the Association Between Survey Wave and Probable Mental Health Problems.**

| **IV** | **Mental Health** | | **Effect Ratio** |
| --- | --- | --- | --- |
|  | **B** | **95% CIs** |  |
| **Wave (Reference: Wave 7 [2015/2016])** |  |  |  |
| Total effect of Wave (Wave 11 [2019/2020]) | 0·26^*^ | 0·06 to 0·46 |  |
| Direct effect of Wave (Wave 11 [2019/2020]) | 0·20^*^ | 0·00 to 0·41 |  |
| *Indirect effect via perceived discrimination* | *0·05^**^* | *0·02 to 0·08* | *20·5%* |

Note: *n* = 12,615. Model is unadjusted for any covariates.

^*^*p* <·05; ^**^*p* <·01; ^***^*p* <·001.

## G. Individual Reasons for Discrimination


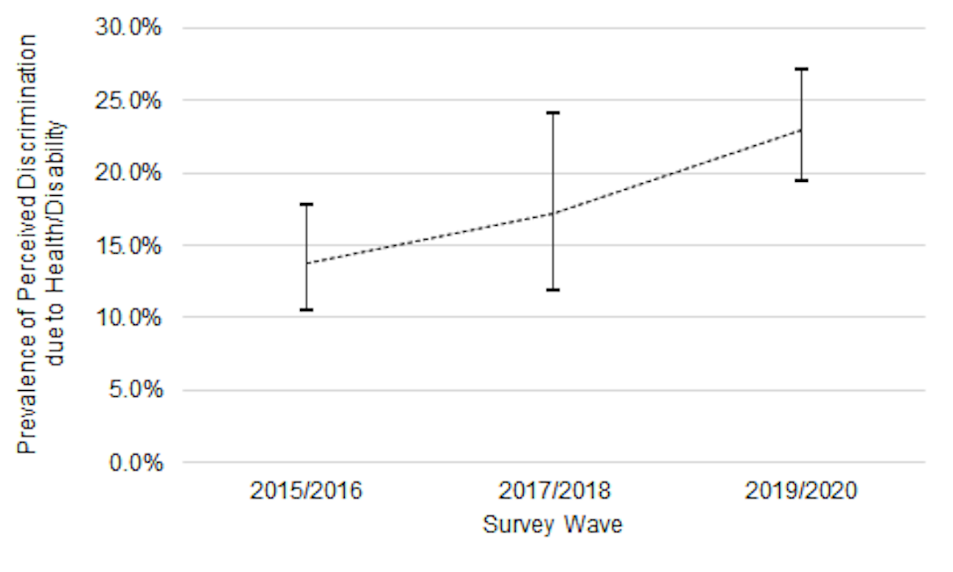

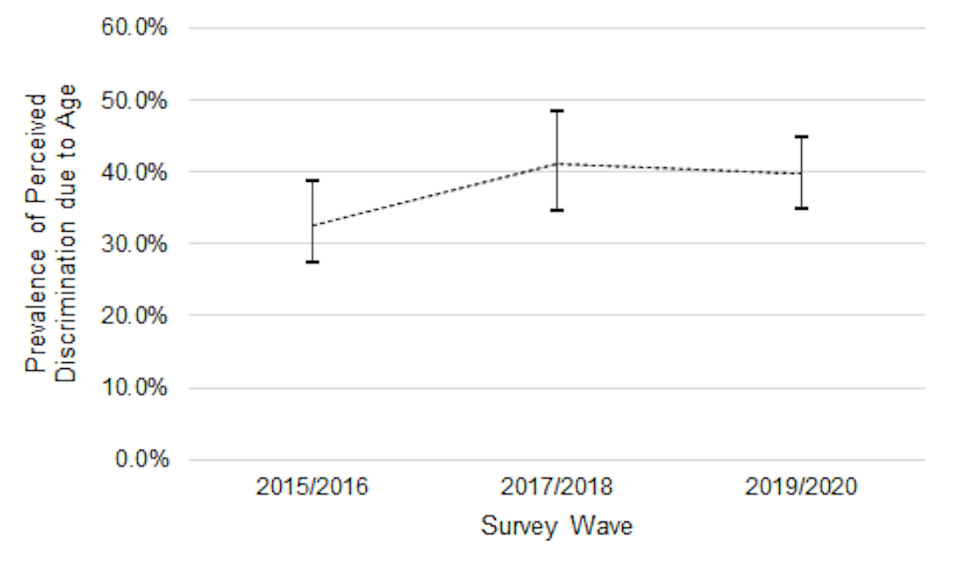

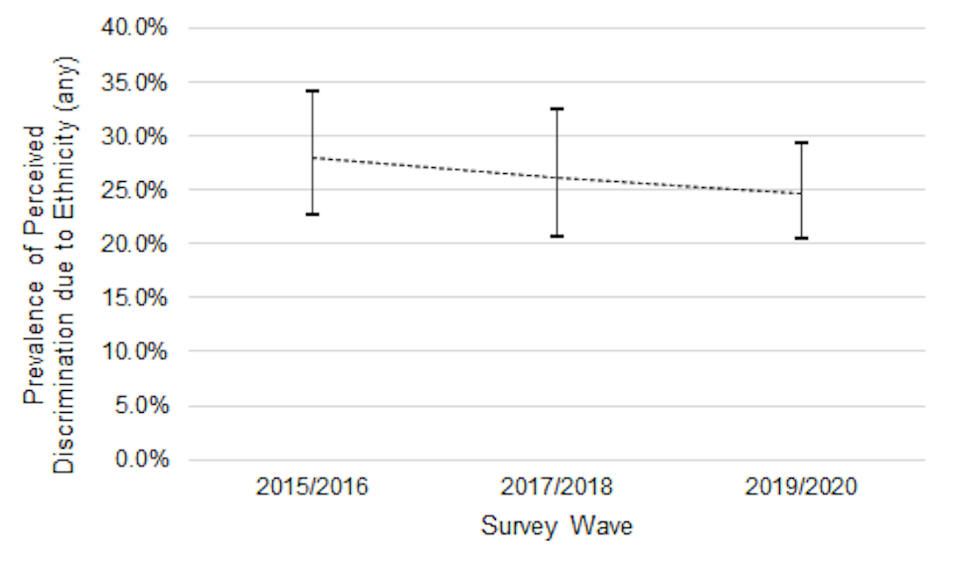

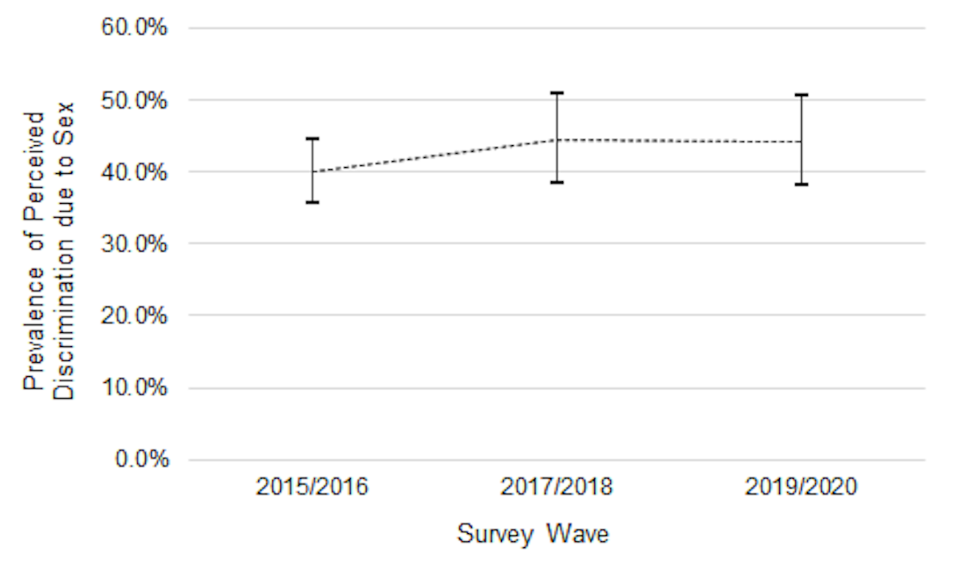


a)

b)

c)

d)

**Figure S2. Weighted Prevalence of Those who Selected Specific Reasons for Their Perceived Discrimination Experience, by Survey Wave.**

a) These are individuals who ticked sex as a reason they perceived discrimination; b) These are individuals who ticked age as a reason they perceived discrimination; c) These are individuals who ticked ethnicity, nationality or language/accent as a reason they perceived discrimination; d) These are individuals who ticked health/disability as a reason they perceived discrimination.

**Table S9. Differences between Sociodemographic Groups in the Changes in Individual Reasons for Perceived Discrimination from 2015/2016 to 2019/2020.**

| **Population Subgroup** | **Difference between Subgroups in the Changes in Individual Reasons for Perceived Discrimination from 2015/2016 to 2019/2020** | |
| --- | --- | --- |
|  | **%** | **(95% CI)** |
| **Sex (Comparison: Males)^a^** |  | |
| Females | -1·1 | (-10·1 to 7·9) |
| **Age Group (Comparison: 16-34 Years)^b^** |  | |
| 35-50 Years | +6·7 | (-16·0 to 29·3) |
| 51-64 Years | -2·2 | (-23·7 to 19·3) |
| 65+ Years | -15·6 | (-38·0 to 6·8) |
| **Ethnic Group (Comparison: White Respondents)^c^** |  | |
| Mixed | +25·2 | (2·5-47·8) |
| Asian | -6·8 | (-28·8 to 15·3) |
| Black | +15·5 | (-14·5 to 45·5) |
| Other | -2·0 | (-53·2 to 49·1) |
| **Health (Comparison: Does not have a longstanding illness/disability)^d^** |  | |
| Has a longstanding illness/disability | +3·8 | (-6·7 to 14·4) |

Note: Interaction terms were added to multivariable models containing all sociodemographic characteristics as covariates. *P*-values are adjusted using the Benjamini-Hochberg method. Differences displayed are lincom differences between the changes in predicted probabilities between waves (marginal effects), multiplied by 100 to get percentage point differences. The difference in the changes are between the displayed comparison group and non-comparison group (e.g. males vs. females). Negative values indicate that the change for the comparison group was greater than the change for the non-comparison group. For details of the changes in the outcomes between waves by subgroup, see Table S10.

^a^Model outcome is perceived discrimination due to sex.

^b^Model outcome is perceived discrimination due to age.

^c^Model outcome is perceived discrimination due to ethnicity (any).

^d^Model outcome is perceived discrimination due to health/disability.

**Table S10. Prevalence of Individual Reasons for Perceived Discrimination by Sociodemographic Subgroup and Survey Wave, and Percentage Point Changes Between 2015/2016 to 2019/2020.**

| **Population Subgroup** | **Individual Reason for Perceived Discrimination^a^** | | | | |
| --- | --- | --- | --- | --- | --- |
|  | **Wave 7**  **(2015/2016)** | **Wave 9**  **(2017/2018)** | **Wave 11**  **(2019/2020)** | **Change between 2015/2016 – 2019/2020^b^** | |
|  | **Weighted *n* & %** | **Weighted *n* & %** | **Weighted *n* & %** | **%** | **(95% CI)** |
| **Overall due to Sex** | **1681**  40·0% | **1606**  44·6% | **1400**  44·2% | +2·3 | (-3·4 to 7·9) |
| **Overall due to Age** | **1681**  32·7% | **1606**  41·2% | **1400**  39·7% | +4·7 | (-3·3 to 12·7) |
| **Overall due to Ethnicity (any)** | **1681**  28·0% | **1606**  26·2% | **1400**  24·6% | -0·4 | (-7·0 to 6·2) |
| **Overall due to Health/Disability** | **1681**  13·7% | **1606**  17·2% | **1400**  23·0% | +9·8^**^ | (3·4-16·3) |
| **Sex^c^** | **1680** | **1606** | **1400** |  | |
| Male | 0·6% | 2·3% | 2·5% | +3·3^*^ | (1·2-5·4) |
| Female | 59·1% | 67·4% | 66·6% | +2·2 | (-6·5 to 11·0) |
| **Age^d^** | **1681** | **1606** | **1400** |  | |
| 16-34 | 31·3% | 43·6% | 35·1% | +6·7 | (-6·1 to 19·5) |
| 35-50 | 8·5% | 7·9% | 20·8% | +13·3 | (-4·7 to 31·4) |
| 51-64 | 33·9% | 48·5% | 40·0% | +4·5 | (-13·9 to 22·9) |
| 65+ | 77·8% | 71·1% | 67·9% | -8·9 | (-26·7 to 8·8) |
| **Ethnic Group^e^** | **1663** | **1591** | **1389** |  | |
| White | 19·0% | 17·6% | 15·1% | -2·1 | (-10·2 to 5·9) |
| Mixed | 34·6% | 62·2% | 54·5% | +23·0 | (2·2-43·9) |
| Asian | 65·4% | 60·5% | 56·2% | -8·9 | (-29·2 to 11·4) |
| Black | 44·0% | 40·7% | 56·5% | +13·3 | (-15·8 to 42·4) |
| Other | 58·5% | 44·6% | 51·4% | -4·2 | (-54·9 to 46·5) |
| **Health (Has longstanding illness/disability)^f^** | **1676** | **1603** | **1391** |  | |
| Yes | 28·6% | 36·5% | 37·6% | +12·0 | (1·8-22·2) |
| No | 2·6% | 3·7% | 9·1% | +8·1 | (1·9-14·3) |

Note: ^a^four reasons are explored as outcomes within the table, those who ticked they perceived discrimination and identified sex as a reason, those who ticked they perceived discrimination and identified age as a reason, those who ticked they perceived discrimination and identified any ethnicity reasons (ethnicity, nationality, or language/accent), and those who ticked they perceived discrimination and identified health/disability as a reason.

^b^Change between 2015/2016 – 2019/2020 is the change in the predicted probability of perceiving discrimination due to the individual reason. Interaction terms were added to multivariable models containing all sociodemographic characteristics as covariates. Differences displayed are lincom differences in predicted probabilities (marginal effects), multiplied by 100 for percentage point differences. Note reported change values differ to the changes in weighted prevalence only, as these change values are from predicted probabilities from an adjusted model. *P*-values are adjusted using the Benjamini-Hochberg method. Weighted *n* = 4199.

^c^Changes by sex are explored for the outcome of perceived discrimination due to sex.

^d^Changes by age are explored for the outcome of perceived discrimination due to age.

^e^Changes by ethnic group are explored for the outcome of perceived discrimination due to ethnicity (any).

^f^Changes by health are explored for the outcome of perceived discrimination due to health/disability.

^*^*p* <·05; ^**^*p* <·01; ^***^*p* <·001.

## H. Impact of COVID-19

**Table S11. Sample Prevalence and Prevalence of Perceived Discrimination and Probable Mental Health Problems in Wave 11 (2019/2020), by Interview Start Date.**

| **Interview Start Date** | **Weighted %^a^** | **Weighted Prevalence** | |
| --- | --- | --- | --- |
|  |  | **Perceived Discrimination^b^** | **Probable Mental Health Problems^c^** |
| Before COVID-19 | 53·0% | 21·0% | 20·7% |
| After COVID-19 | 47·0% | 19·1% | 23·7% |

^a^Weighted *n* = 6859. Unweighted *n* = 7132. Unweighted % = 43·3% Before COVID-19, 56·7% After COVID-19.

^b^Weighted *n* = 6757.

^c^Weighted *n* = 6377.

**Changes in Perceived Discrimination**

We repeated primary discrimination analysis, while additionally controlling for the impact of COVID-19 (interview date). Exploring only participants in 2019/2020, we found no significant difference in perceived discrimination for those starting their interview prior to, compared to post COVID-19 (-2·1%, 95% CI: -7·2 to 3·1, *p* =·429). Consistent with the main analysis (2015/2016 vs 2019/2020), perceived discrimination increased significantly by 6·9% between waves (95% CI: 3·5-10·4, *p* <·001).

**Changes in Probable Mental Health Problems**

We repeated primary probable mental health problems analysis, while additionally controlling for the impact of COVID-19 (interview date). Exploring only participants in 2019/2020, we found a significant difference in probable mental health problems of 5·5% for those starting their interview after the COVID-19 outbreak, compared to prior to this (95% CI: 0·4-10·7, *p* =·036). Unlike the main analysis (2015/2016 vs 2019/2020), probable mental health problems no longer significantly changed between waves (+2·1%, 95% CI: -1·7 to 5·9, *p* =·282).

**Differences between Sociodemographic Groups in the Changes in Perceived Discrimination and Probable Mental Health Problems**

After additionally controlling for COVID-19 (interview start date), we found that consistent with the main analysis there was no statistical evidence that survey wave interacted with any of the sociodemographic variables for perceived discrimination or probable mental health problems (see Table S12), indicating that changes from 2015/2016 to 2019/2020 were not meaningfully different across sociodemographic groups.

**Table S12. Differences between Sociodemographic Groups in the Changes in Perceived Discrimination and Probable Mental Health Problems from 2015/2016 to 2019/2020, after Adjusting for Interview Start Date (COVID-19 Impact).**

| **Population Subgroup** | **Differences between Subgroups in the Changes in Perceived Discrimination from 2015/2016 to 2019/2020^a^** | | **Difference between Subgroups in the Changes in Probable Mental Health Problems from 2015/2016 to 2019/2020^b^** | |
| --- | --- | --- | --- | --- |
|  | **%** | **(95% CI)** | **%** | **(95% CI)** |
| **Sex (Comparison: Males)** |  | |  | |
| Females | +2·1 | (-3·8 to 8·1) | +3·4 | (-3·0 to 9·8) |
| **Age Group (Comparison: 16-34 Years)** |  | |  | |
| 35-50 Years | -4·5 | (-11·3 to 2·3) | +6·3 | (-4·7 to 17·3) |
| 51-64 Years | +4·5 | (-3·4 to 12·4) | +4·2 | (-5·2 to 13·6) |
| 65+ Years | -1·8 | (-9·9 to 6·3) | +2·9 | (-5·8 to 11·5) |
| **Ethnic Group (Comparison: White Respondents)** |  | |  | |
| Mixed | -1·9 | (-19·0 to 15·3) | +1·0 | (-12·6 to 14·6) |
| Asian | +1·9 | (-6·3 to 10·1) | -5·5 | (-13·1 to 2·1) |
| Black | -9·9 | (-22·5 to 2·6) | -9·6 | (-19·0 to -0·1) |
| Other | -10·9 | (-26·5 to 4·7) | -9·1 | (-35·3 to 17·1) |
| **Religious (Comparison: Does not identify as belonging to a religion)** |  | |  | |
| Identifies as belonging to a religion | -3·5 | (-9·5 to 2·6) | -3·0 | (-10·0 to 4·0) |
| **Health (Comparison: Does not have a longstanding illness/disability)** |  | |  | |
| Has a longstanding illness/disability | +5·3 | (-2·2 to 12·8) | -0·5 | (-7·6 to 6·7) |
| **Equivalised Household Income Per Month (Comparison: Category 1)** |  | |  | |
| Category 2 | -11·6 | (-21·9 to -1·3) | -1·5 | (-13·6 to 10·6) |
| Category 3 | -4·8 | (-16·5 to 6·8) | -3·0 | (-14·6 to 8·6) |
| Category 4 | +2·5 | (-7·7 to 12·7) | +4·6 | (-6·3 to 15·5) |
| Category 5 | +0·6 | (-9·8 to 11·0) | +0·4 | (-9·4 to 10·2) |
| **Education Level (Comparison: Other/No Qualification)** |  | |  | |
| High School Qualification | +6·9 | (-2·2 to 16·0) | +1·5 | (-7·1 to 10·2) |
| University Degree | +7·0 | (-1·8 to 15·8) | +6·0 | (-2·6 to 14·6) |
| **Current Job (Comparison: Not in Paid Employment in the Last Week)** |  | |  | |
| Routine | +0·5 | (-8·3 to 9·2) | +0·8 | (-9·2 to 10·7) |
| Intermediate | -2·4 | (-10·5 to 5·6) | +1·9 | (-6·7 to 10·6) |
| Management & Professional | +4·5 | (-2·5 to 11·5) | +8·7 | (1·2-16·3) |

Note: Interaction terms were added to multivariable models containing all sociodemographic characteristics and interview start date (COVID-19) as covariates. *P*-values are adjusted using the Benjamini-Hochberg method. Differences displayed are lincom differences between the changes in predicted probabilities between waves (marginal effects), multiplied by 100 to get percentage point differences. The difference in the changes are between the displayed comparison group and non-comparison group (e.g. males vs. females). Negative values indicate that the change for the comparison group was greater than the change for the non-comparison group. For details of the changes in the outcomes between waves by subgroup, see Table S13.

^a^Perceived discrimination weighted *n* = 14,172.

^b^Probable mental health problems weighted *n* = 13,713.

**Changes by Population Subgroup**

For details of the specific changes for each subgroup, see Table S13. COVID-19 adjusted changes in perceived discrimination between 2015/2016 to 2019/2020 were largely consistent with the main analysis. However, unlike the main analysis, no significant subgroup changes in probable mental health problems were found after adjusting for COVID-19.

**Table S13. Percentage Point Changes in Perceived Discrimination and Probable Mental Health Problems Between 2015/2016 and 2019/2020, by Population Subgroup after Controlling for Interview Start Date (COVID-19 Impact).**

| **Population Subgroup** | **Change between 2015/2016 – 2019/2020^a^** | | | |
| --- | --- | --- | --- | --- |
|  | **Perceived Discrimination^b^** | | **Probable Mental Health Problems^c^** | |
|  | **%** | **(95% CI)** | **%** | **(95% CI)** |
| **Overall** | +6·9^***^ | (3·5-10·4) | +2·1 | (-1·7 to 5·9) |
| **By Sex** |  | |  | |
| Males | +6·0^**^ | (2·3-9·6) | +0·5 | (-4·5 to 5·5) |
| Females | +8·1^*^ | (2·6-13·6) | +3·9 | (-1·1 to 8·8) |
| **By Age** |  | |  | |
| 16-34 | +7·3 | (0·9-13·8) | -1·3 | (-9·5 to 6·9) |
| 35-50 | +2·8 | (-2·4 to 8·0) | +5·0 | (-3·0 to 13·0) |
| 51-64 | +11·9^**^ | (5·9-17·9) | +2·9 | (-2·7 to 8·5) |
| 65+ | +5·5 | (0·3-10·8) | +1·6 | (-3·2 to 6·4) |
| **By Ethnic Group** |  | |  | |
| White | +7·5^**^ | (3·9-11·1) | +3·0 | (-1·3 to 7·2) |
| Mixed | +5·7 | (-11·5 to 22·8) | +4·0 | (-9·3 to 17·2) |
| Asian | +9·4 | (1·1-17·7) | -2·6 | (-9·7 to 4·6) |
| Black | -2·4 | (-14·9 to 10·1) | -6·6 | (-15·9 to 2·8) |
| Other | -3·4 | (-19·0 to 12·2) | -6·2 | (-3·2 to 19·3) |
| **By Religious** |  | |  | |
| Identifies as belonging to a religion | +5·2^* +^ | (1·1-9·4) | +0·6 | (-4·3 to 5·4) |
| Does not identify as belonging to a religion | +8·7^**^ | (3·6-13·7) | +3·6 | (-1·9 to 9·1) |
| **By Health** |  | |  | |
| Has a longstanding illness/disability | +10·4^*^ | (3·9-16·8) | +1·8 | (-4·8 to 8·4) |
| Does not have a longstanding illness/disability | +5·0^*^ | (1·0-9·1) | +2·3 | (-1·9 to 6·4) |
| **By Equivalised Household Income Per Month** |  | |  | |
| Category 1 (lowest) | +9·3 | (0·9-17·7) | +1·7 | (-6·9 to 10·3) |
| Category 2 | -2·3 | (-8·4 to 3·8) | +0·2 | (-7·9 to 8·4) |
| Category 3 | +4·4 | (-4·2 to 13·1) | -1·3 | (-9·1 to 6·5) |
| Category 4 | +11·8^**^ | (6·1-17·5) | +6·3 | (-0·5 to 13·1) |
| Category 5 (highest) | +9·9^*^ | (3·4-16·4) | +2·1 | (-4·2 to 8·4) |
| **By Education Level** |  | |  | |
| Other/No Qualification | +1·0 | (-7·2 to 9·2) | -1·4 | (-9·1 to 6·4) |
| High School Qualification | +7·9^**^ | (3·1-12·6) | +0·2 | (-5·4 to 5·7) |
| University Degree | +8·0^**^ | (3·2-12·7) | +4·6 | (-0·2 to 9·4) |
| **By Current Job** |  | |  | |
| Not in paid employment in the last week | +5·9 | (0·9-11·0) | -1·0 | (-7·3 to 5·4) |
| Routine | +6·4 | (-1·0 to 13·8) | -0·2 | (-8·0 to 7·6) |
| Intermediate | +3·5 | (-3·1 to 10·1) | +1·0 | (-5·5 to 7·4) |
| Management & Professional | +10·5^**^ | (4·6-16·4) | +7·8 | (1·6-13·9) |

Note: ^a^Change between 2015/2016 – 2019/2020 is the change in the predicted probability of perceiving discrimination or experiencing probable mental health problems. Interaction terms were added to multivariable models containing all sociodemographic characteristics and interview start date (COVID-19) as covariates. Differences displayed are lincom differences in predicted probabilities (marginal effects), multiplied by 100 for percentage point differences. Reported *p*-values of explored interaction terms are adjusted using the Benjamini-Hochberg method.

^b^Perceived discrimination weighted *n* = 14,172.

^c^Probable mental health problems weighted *n* = 13,713.

^+^ Significant after controlling for COVID-19.

^*^ *p* <·05; ^**^ *p* <·01; ^***^ *p* <·001.

**Association Between Perceived Discrimination and Probable Mental Health Problems**

After additionally adjusting for COVID-19 (interview date), we found that consistent with the main results, probable mental health problems were significantly greater by 11·6% (95% CI: 7·1-16·2, *p* <·001) for those who had perceived discrimination, compared to those who had not. We explored an interaction between perceived discrimination and survey wave (2015/2016 vs 2017/2018) for the prediction of probable mental health problems in a model that controlled for all sociodemographic characteristics and COVID-19 (interview date). This revealed that the size of the change in probable mental health problems between 2015/2016 and 2019/2020 was not meaningfully different for those who did and did not perceive discrimination (difference in the change = +5·0%, 95% CI: -3·6 to 13·6, *p* =·253). Specifically, those who had perceived discrimination had a 5.2% non-significant increase in probable mental health problems between 2015/2016 to 2019/2020 (95% CI: -3·0 to 13·3, *p* =·214), and those who had not perceived discrimination had a non-significant 0.1% increase between waves (95% CI: -4·0 to 4·3, *p* =·949).

Note exploratory mediation models were not conducted, as the survey wave and mental health association (total effect) was non-significant after controlling for interview start date (COVID-19).

## I. Exploratory Analysis: Exploration of Earlier (Pre-Study) Waves of UKHLS

Prior to the beginning of the study period (2015/2016), limits to sampling and UKHLS survey weights did not allow us to estimate representative prevalence of perceived discrimination and probable mental health problems (see Supplementary A for more detail). However, given that main study period trends were consistent in weighted vs. unweighted analyses (see Supplementary C), to better understand historic trends in perceived discrimination and probable mental health problems we examined unweighted changes in early waves of UKHLS from 2009/2010 (wave 1) to 2013/2014 (wave 5). We used unweighted regressions with standard errors clustered by the individual identifier to account for the repeated cross-sectional design. All sociodemographic characteristics are included as covariates. For each comparison, only the waves explored are included within the sample (e.g. for 2009/2010 to 2011/2012, only participants from these waves are included within the analysis). Figure S3 displays unweighted trends including study waves prior to the primary analysis. This shows that prior to 2015/2016, perceived discrimination and probable mental health problems had been on a downward trend (compared to 2009/2010). For significance of trends, see Table S14.

**Figure S3. Unweighted Prevalence of Perceived Discrimination and Probable Mental Health Problems Including Earlier Survey Waves in UKHLS.**


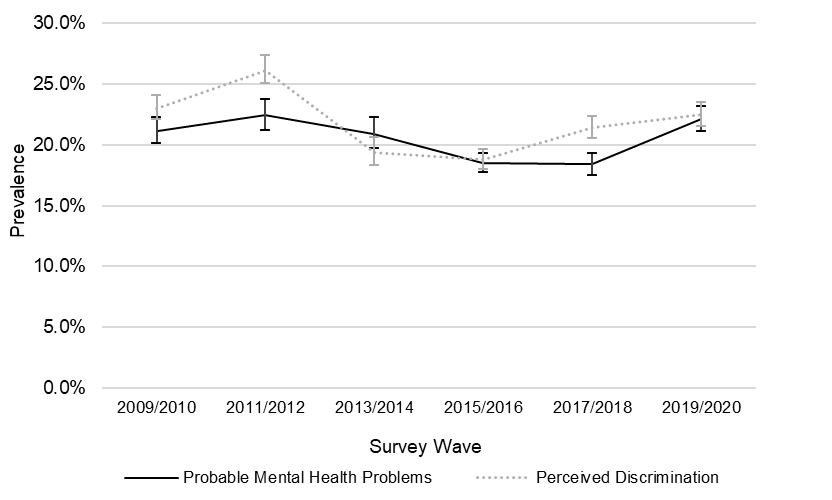


Primary Analysis Study Waves

***

***

***

*

*

***

Earlier (Pre-Study) UKHLS Waves

*

Note: 2015/2016 line denotes where the primary analysis study period begins. Significance of the change between waves for perceived discrimination is indicated above the graph line, and for probable mental health problems it is indicated below the graph line. Significance of each change is determined through unweighted models controlling for all sociodemographic characteristics (see Table S14).

^*^*p* <·05; ^**^*p* <·01; ^***^*p* <·001.

**Table S14. Unweighted Percentage Point Changes in Perceived Discrimination and Probable Mental Health Problems Between Each Survey Wave from 2009/2010 to 2019/2020.**

| **Change between Survey Waves^a^** | **Perceived Discrimination** | | | **Probable Mental Health Problems** | | |
| --- | --- | --- | --- | --- | --- | --- |
|  | **Sample *n*** | **%** | **(95% CI)** | **Sample *n*** | **%** | **(95% CI)** |
| 2009/2010 to 2011/2012^b^ | 13,329 | +2·8^***^ | (1·4-4·1) | 10,019 | +1·8^*^ | (0·4-3·3) |
| 2011/2012 to 2013/2014^b^ | 10,073 | -6·4^***^ | (-7·8 to -4·9) | 8,365 | -1·0 | (-2·5 to 0·5) |
| 2013/2014 to 2015/2016^b^ | 13,023 | +0·4 | (-0·9 to 1·7) | 12,021 | -1·8^*^ | (-3·2 to -0·4) |
| 2015/2016 to 2017/2018 | 15,199 | +2·1^***^ | (1·0-3·3) | 14,463 | -0·7 | (-1·8 to 0·4) |
| 2017/2018 to 2019/2020 | 12,916 | +1·4^*^ | (0·2-2·7) | 12,118 | +4·1^***^ | (2·8-5·3) |

Note: ^a^Change between survey waves is the change in the predicted probability of perceiving discrimination or experiencing probable mental health problems between the survey wave dates. All sociodemographic characteristics are included as covariates. Differences displayed are lincom differences in predicted probabilities (marginal effects), multiplied by 100 for percentage point differences.

^b^Difference between waves prior to the primary study period.

^*^ *p* <·05; ^**^ *p* <·01; ^***^ *p* <·001.
